# Dietary patterns, brain morphology and cognitive performance in children: Results from a prospective population-based study

**DOI:** 10.1007/s10654-023-01012-5

**Published:** 2023-05-08

**Authors:** Yuchan Mou, Elisabet Blok, Monica Barroso, Pauline W. Jansen, Tonya White, Trudy Voortman

**Affiliations:** 1 Department of Epidemiology, Erasmus MC, University Medical Center, Rotterdam, the Netherlands; 2 The Generation R Study Group, Erasmus MC, University Medical Center, Rotterdam, the Netherlands; 3 Department of Child and Adolescent Psychiatry/Psychology, Erasmus MC, University Medical Center, Rotterdam, the Netherlands; 4 Department of Psychology, Education and Child Studies, Erasmus University Rotterdam, Rotterdam, the Netherlands; 5 Department of Radiology and Nuclear Medicine, Erasmus MC, University Medical Center, Rotterdam, the Netherlands; 6 Section on Social and Cognitive Developmental Neuroscience, National Institutes of Mental Health, Bethesda, MD USA

**Keywords:** Dietary patterns, Childhood, MRI, Brain volume, Hippocampus, Amygdala, Cortical thickness, Cognitive function, Intelligence quotient

## Abstract

Dietary patterns in childhood have been associated with child neurodevelopment and cognitive performance, while the underlying neurobiological pathway is unclear. We aimed to examine associations of dietary patterns in infancy and mid-childhood with pre-adolescent brain morphology, and whether diet-related differences in brain morphology mediate the relation with cognition. We included 1888 and 2326 children with dietary data at age one or eight years, respectively, and structural neuroimaging at age 10 years in the Generation R Study. Measures of brain morphology were obtained using magnetic resonance imaging. Dietary intake was assessed using food-frequency questionnaires, from which we derived diet quality scores based on dietary guidelines and dietary patterns using principal component analyses. Full scale IQ was estimated using the Wechsler Intelligence Scale for Children-Fifth Edition at age 13 years. Children with higher adherence to a dietary pattern labeled as ‘Snack, processed foods and sugar’ at age one year had smaller cerebral white matter volume at age 10 (B = -4.3, 95%CI -6.9, -1.7). At age eight years, higher adherence to a ‘Whole grains, soft fats and dairy’ pattern was associated with a larger total brain (B = 8.9, 95%CI 4.5, 13.3), and larger cerebral gray matter volumes at age 10 (B = 5.2, 95%CI 2.9, 7.5). Children with higher diet quality and better adherence to a ‘Whole grains, soft fats and dairy’ dietary pattern at age eight showed greater brain gyrification and larger surface area, clustered primarily in the dorsolateral prefrontal cortex. These observed differences in brain morphology mediated associations between dietary patterns and IQ. In conclusion, dietary patterns in early- and mid-childhood are associated with differences in brain morphology which may explain the relation between dietary patterns and neurodevelopment in children.

**Supplementary Information:**

The online version contains supplementary material available at 10.1007/s10654-023-01012-5.

## Introduction

Diet is an important modifiable factor that modulates brain development by supplying energy and nutrients. Previous studies have established that poor diet quality during the fetal and early postnatal period impacts neurodevelopment in early life due to the increased risk of essential nutrient deficiency or protein-energy malnutrition [1–3]. Beyond nutrition in early life, children develop distinct dietary patterns as they grow. In the meantime, structural and functional brain plasticity continues to develop throughout childhood and adolescence [4]. Thus, the brain remain vulnerable to poor nutrition during this period of ongoing growth and modeling.

As a complement to the evidence regarding nutrients and neurodevelopment, there has been increasing interest to the relationship between overall dietary patterns and children's brain development. Dietary patterns, which can be quantified by predefined indices (e.g., diet quality score) or derived empirically from dietary data, are closely related to eating behaviors in the real-world setting by considering how foods and nutrients are combined [5], and presenting the ability to detect the variation of dietary composition and its effect on health. Examples that are often studied are: prudent or healthy dietary patterns, often featured as high whole grains, fruits and vegetables intake; western-like or unhealthy dietary patterns, characterized by high intakes of refined grains, saturated fat, and sugar. Recent studies have shown that dietary patterns are established in early childhood and remain relatively stable into adulthood, with less healthy patterns showing the strongest stability [6–8]. With ongoing global nutrition transitioning towards unhealthy diets [9], more than half of children have poor-quality diets in low-, middle-, and high-income countries [10–13], raising concerns about its cascading effect on children’s brain development.

A growing body of research has identified consistent temporal associations between children’s dietary patterns and cognitive performance in cohort studies. For instance, a better diet quality [14] or a prudent dietary pattern at different ages through childhood is linked to higher intelligence quotient (IQ) scores of children and adolescents [15], even when accounting for socioeconomic status and home environment. In contrast, children and adolescents with higher adherence to a western-like dietary pattern showed diminished cognitive functioning and lower IQ scores [16, 17]. Among the complex mechanisms underlying the relation between diet and cognitive performance, structural brain alteration may be a detectable neurobiological marker on the pathway. Accumulating evidence from observational studies suggests that a Mediterranean diet, which shows neurocognitive protective effects, is associated with global brain morphological alteration in adults, including total brain volume, gray and white matter volume, as well as cortical thickness [18]. Although studies among children are sparse, neuroimaging studies have identified positive links between IQ and alterations in both global brain volumetric measures in children using structural magnetic resonance imaging (MRI) [19, 20]. Together with evidence on the effect of several individual micronutrients [21] and macronutrients [22] on the brain, global brain morphological changes may also underlie the associations between dietary patterns and cognition in children. However, there is a lack of knowledge as to whether dietary patterns in children are associated with global brain morphology and whether brain morphology underlies the associations between dietary patterns and cognitive performance.

Dietary effects on neurodevelopment may also be specific to certain brain regions. The hippocampus and amygdala may be particularly sensitive to the effects of specific dietary patterns, such as high-fat diet diets. The hippocampus is involved in integrating multimodal information and is critical for memory, learning, and is also involved in appetitive, digestive and learned eating behaviors [23]. The amygdala has been implicated in feeding, the reward systems associated with eating, and modulating food consumption [24]. Animal studies have found that a western diet has adverse effects on hippocampal dependent memory function and neurogenesis by triggering metabolic alterations [25]. Additionally, western diets also induce inflammation in the hippocampus [26, 27], and enhance amygdala-dependent, cue-based memory in juvenile mice [28]. As a result of adapting to diet-induced endocrine abnormalities, structural alteration may occur in the hippocampus and amygdala. Moreover, structural changes may also be a neurobiological marker of functional disruption. A longitudinal study of seniors has found that individuals with higher adherence to a Western-style diet had decreased left hippocampal volumes four years later [29]. It is, however, unclear whether different dietary patterns are associated with the volumetric differences of the hippocampus and amygdala in children. Only one recent study in children investigated the effect of a western diet on hippocampal and amygdala volume, but did not observe an association. However, they found that increased fat consumption was related to a smaller left hippocampal volume [30]. This study has a small sample size and the western diet was calculated by only combining percentage of daily calories from fat and sugar, which may not capture the Western dietary pattern consumed in the general population.

Within this context, we aimed to examine the prospective association of dietary patterns in infancy and mid-childhood with brain morphology assessed at the age of 10 years in a large population-based cohort, and to examine whether dietary pattern-related differences in brain morphology mediate associations of dietary pattern adherence with full scale IQ at age 13 years. Based on evidence from animal studies, we examined the relationship between dietary patterns and two regional brain volumes (hippocampus and amygdala). Due to the paucity of information on the association of overall diet with brain morphology in the literature, we used an exploratory approach that involves global volumetric measures (total brain, cerebral white matter, cerebral gray matter), and subcortical volumetric measures (hippocampus and amygdala). In addition, we studied surface-based brain measures, which provide a different approach to evaluate the overall cortex, serving as exploratory analyses of whole brain measurements of gyrification, surface area and cortical thickness. We used two approaches to define dietary patterns: a priori*-*defined diet quality scores based on national dietary guidelines, and a posteriori-derived dietary patterns using principal components in the population. For global and subcortical volumetric measures, we hypothesized that higher adherence to a western-like dietary pattern and lower diet quality score would be associated with smaller global brain volumes at 10 years of age, and would be associated with smaller hippocampal and amygdala volume. We expected that positive associations would be found for a prudent dietary pattern or a better diet quality. For surface-based measures, we did not have prior hypotheses. Moreover, we expected that differences in brain morphology in relation to dietary pattern adherence would mediate the relationship between dietary pattern adherence and full-scale IQ. The findings of this study will facilitate future hypotheses on overall diet and brain development, as this has yet to be studied in children in a population-based setting.

## Methods

### Study design and population

This study was embedded in The Generation R Study, an ongoing population-based prospective cohort from early fetal life onward in Rotterdam, the Netherlands. The detailed information on the general design of the Generation R Study has been described in detail elsewhere [31]. Of 9749 eligible pregnant women with a delivery date between April 2002 to January 2006, 7893 mother–child dyads consented for the postnatal follow-up. The study was approved by the Medical Ethics Committee of Erasmus Medical Center, Rotterdam. Written informed consent was obtained from all participating children and their parents.

**Fig. 1:**
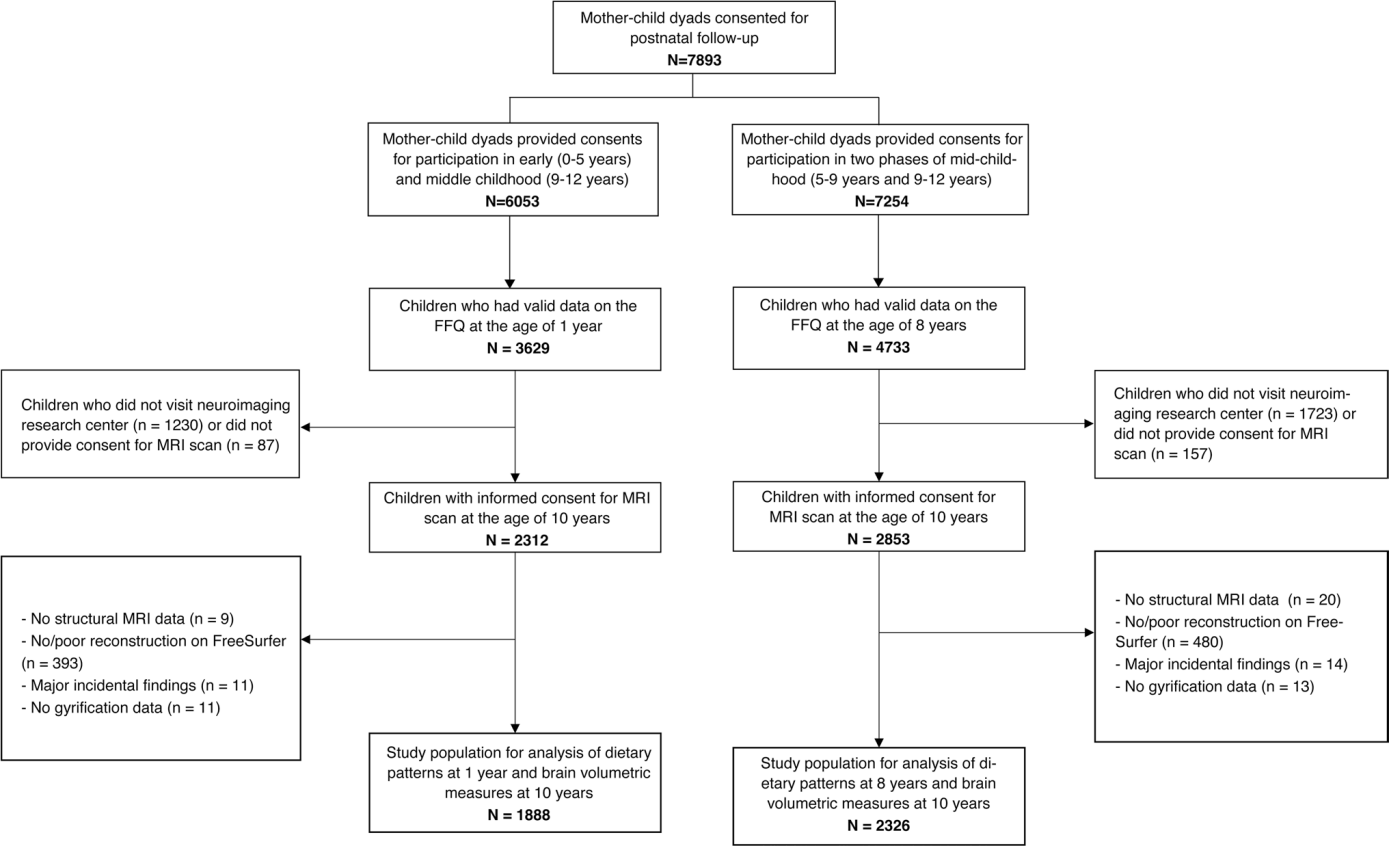
Study population flow chart

Dietary information was obtained using validated food frequency questionnaires (FFQs), which were completed, generally by the mothers, for 3629 children at one year-of-age (out of 5088 mothers who received the FFQ when children were one year old, response rate: 71.3%) [32] and for 4733 children at eight years-of-age (out of 7662 mothers who received the FFQ when children were eight years old, response rate: 61.8%) [12]. Structural T_1_-weighted images were obtained when children were 10 years [33]. We excluded children who did not visit the neuroimaging research center, or did not provide consent for the MRI scan. For those with valid dietary data at age one year, images were available for 2312 children, and for those with dietary data at eight years of age, images were available for 2853 children. Further, children were excluded if images could not be reconstructed, had poor quality, if major incidental findings were found, or if the gyrification index could not be calculated. The final study population comprised 1888 children for analysis of diet at age one year and 2326 children for analysis of diet at age eight years (Fig. 1).

### Dietary assessment

Dietary intake was assessed when children were approximately one year-of-age (median 12.9 months, IQR: 12.7, 13.9) and eight years-of-age (median 8.2 years, IQR: 8.0, 8.2) using two age-specific validated semi-quantitative FFQs. The FFQs included food items based on foods that were frequently consumed by 9–18 months-old and 2-to-12 year-old children in national food consumption surveys in the Netherlands [34, 35], resulting in 221 items for age one year and 71 items for age eight years. Each FFQ contains questions on frequency of consumption, portion size, the types of food items and corresponding food preparation methods over the past 4 weeks. The information was converted into grams of individual food items per day based on standardized portion sizes using SAS VoVris (Vovris V2.4, TNO, 1999–2006). Total energy and nutrient intake were calculated using reference data from the Dutch Food Composition Tables (NEVO 2001). The FFQ at age 1 year was evaluated for nutrient intakes against three 24-h recalls in a representative sample of 32 Dutch children aged 14 months (*r* ranging from 0.36 to 0.74) [32]. The FFQ at age 8 years was validated for energy intake (*r* = 0.62) using the doubly labeled water method among 4 to 6 years Dutch children (n = 30) [36].

### Dietary patterns

Both a priori and a posteriori methods were used to evaluate dietary patterns. A priori-defined Diet-Quality Scores (DQS) at ages one and eight years were quantified on the basis of Dutch dietary guidelines [12, 32]. Adherence to recommendations for ten food groups were considered at each age, with intake cut-offs based on age-specific dietary recommendations. Specifically, DQS at one year-of-age (DQS-1y) includes scores for vegetables (≥ 100 g/d); fruit (≥ 150 g/d); bread and cereals (≥ 70 g/d); rice, pasta, potatoes, and legumes (≥ 70 g/d); dairy (≥ 350 g/d); meat, poultry, eggs, and meat substitutes (≥ 35 g/d); fish (≥ 15 g/d); oils and fats (≥ 25 g/d); candy and snacks (≤ 20 g/d); and sugar-sweetened beverages (≤ 100 g/d) [32]. DQS for children at eight years-of-age (DQS-8y) includes fruit (≥ 150 g/d); vegetables (≥ 150 g/d); whole grains (≥ 90 g/d); fish (≥ 60 g/wk); legumes (≥ 84 g/wk); nuts (≥ 15 g/d); dairy (≥ 300 g/d); oils and soft or liquid margarines (≥ 30 g/d); sugar-containing beverages (≤ 150 g/d); and high-fat and processed meat (≤ 250 g/wk) [12]. To determine the DQS for each participant, the ratio of reported and recommended intake for each food group was first calculated. Then a summary score was calculated by summing the individual food group scores. The DQSs range from 0 to 10, with higher scores reflecting a higher adherence to the dietary guidelines. Both DQS-1y and DQS-8y were validated for intake of macronutrients and micronutrients among children participating in the Generation R Study [12, 32].

A posteriori*-*derived dietary patterns were extracted using principal components analysis (PCA). Food items from the FFQ at age one year were classified into 27 food groups (Supplemental Table 2) in line with previous studies [37, 38], and food items from the FFQ at age eight years were grouped into 26 food groups (Supplemental Table 3). The varimax rotation method was used to obtain the orthogonal components with the aim of minimizing the covariance between the components. The choice of numbers of dietary patterns to extract was based on the scree plot and Kaiser criterion test. Consequently, the dietary patterns at ages one and eight years with eigenvalues of 1.3 or larger were considered as the most common dietary patterns in the study population. Factor loadings were obtained for each food group, indicating the correlation between the food group and each dietary pattern. Dietary pattern scores for each participant were calculated by summing the food group intake weighted by the corresponding factor loading. The scores were standardized with higher scores reflecting higher similarity to the extracted dietary patterns. Dietary patterns were named after the three food groups with the highest factor loadings.

### Brain morphometry assessments

At the age of 10 years (median 9.9, IQR 9.7, 10.1), the children were invited to the MRI center. Prior to the MRI procedure, the children participated in a mock MRI session. MR images were acquired on a 3.0 Tesla GE Discovery MR750w MRI system (General Electric Healthcare, Milwaukee, WI, USA) scanner using an 8-channel head coil. The high-resolution T_1_-weighted sequence were obtained using a 3D coronal inversion recovery fast spoiled gradient recalled (IR-FSPGR, BRAVO) sequence (TR = 8.77 ms, TE = 3.4 ms, TI = 600 ms, NEX = 1, flip angle = 10°, field of view = 220 × 220 mm, number of slices = 230, in-plane resolution = 1.0 mm^3^) [33]. MRI scans were evaluated by trained researchers and neuroradiologists using a predefined protocol for incidental findings [39]. Children with the presence of pronounced incidental findings were excluded from the study.

Volumetric segmentation and cortical reconstruction were processed using FreeSurfer version 6.0 analysis suite (https://surfer.nmr.mgh.harvard.edu/) with standard processing procedure which have been described previously [40]. Volumes of the right and left hemispheres were summed to obtain global and regional volumes, including total brain, cerebral white and gray matter, and hippocampus and amygdala volumes. For surface-based analyses, cortical thickness, surface area and gyrification were quantified. The quality of cortical reconstructions was visually inspected, and images were removed in case of insufficient quality [40].

### Cognitive performance

Estimated full scale IQ of children was derived from a subset of the Wechsler Intelligence Scale for Children-Fifth Edition (WISC-V) assessed when they were 13–16 years of age. The WISC-V is an instrument assessing individual cognitive functioning in 6- to 16-year-olds. In collaboration with Pearson (Pearson Clinical Assessment, San Antonio, TX, USA), four core subtests were selected from the WISC-V to derive an estimated full scale IQ. The four subtests included vocabulary, matrix reasoning, digit span and coding, corresponding to measurement of verbal comprehension, fluid reasoning, working memory and processing speed separately. All four subtests were administered by trained research assistants. The detailed method of administering the four subtests is described in a previous study [41]. Raw subsets scores were first converted to age-standardized T-scores (ranging from 1 to 19) based on Dutch norm scores, and were summed and converted to a four-subtest estimated full scale IQ.

### Covariates

Several child and maternal characteristics were considered as potential confounders based on prior literature [12, 42–45], and we plotted a simplified DAG to determine a minimal list of confounders to adjust in the model (Supplemental Fig. 1). Child sex and date of birth were derived from medical records filled in by obstetricians and community midwives. Self-reported questionnaires during pregnancy were used to collect information on maternal education and household income, maternal psychopathology symptoms and lifestyle-related factors during pregnancy, including smoking status, alcohol use, and folic acid supplement use. Maternal highest education level was dichotomized into low (ranging from no education up to lower vocational training) and high (higher vocational training/university) educational level. Household income was categorized into < 1200€, 1200—2200€ and > 2200€ per month. Smoking during pregnancy was categorized into never smoking, smoking until pregnancy was known, and continued smoking. Alcohol use during pregnancy was categorized into never drinking, drinking until pregnancy was known and continued drinking. Folic acid use was categorized into no use during embryogenesis, started the first 10 week and started periconceptional. Maternal psychopathology symptoms were assessed at the third trimester by the Brief Symptom Inventory [46], from which the global severity index was calculated. Maternal dietary intake during pregnancy was measured by food frequency questionnaires, from which pre-defined diet quality scores including 15 food components were calculated reflecting adherence to Dutch dietary guidelines, as described in detail elsewhere [47]. Child ethnic background was determined based on the country of birth of the parents, and grouped into Dutch, non-Dutch Western, and non-Dutch non-Western, in line with previous work in the same cohort [48]. Child energy intake was estimated from food frequency questionnaires at ages one and eight years old. Height and weight were measured by trained staff at the research center when children were 10 years old. Child’s sex- and age-specific BMI (kg/m^2^) standard deviation (SD) scores were calculated using Dutch reference growth curves. Intracranial volumes were extracted from FreeSurfer metrics.

### Statistical analysis

Characteristics of the study population were described as mean (SD) for continuous variables with normal distribution, median (IQR) for continuous variables with a skewed distribution, or percentages for categorical variables.

We examined individual associations of a priori and a posteriori dietary patterns with brain morphology using multiple linear regression models. For analysis of global and subcortical brain morphometry, the outcomes of interest were total brain, cerebral white matter, cerebral gray matter, hippocampal and amygdala volumes. Next, the associations of a priori and significant a posteriori dietary patterns with surface-based morphometry, including cortical thickness, surface area and gyrification, were examined. We used two models to examine the associations. Model 1 included child sex and age at neuroimaging assessment. Model 2 was further adjusted for maternal education, household income, child ethnic background, child energy intake, child BMI at the age of 10 years, maternal diet quality, smoking, alcohol use, folic acid use, maternal psychopathology symptoms during pregnancy. For analyses including the hippocampus and amygdala, intracranial volumes were additionally included in model 2. Based on findings from previous studies suggesting that a western diet is associated with left hippocampal volume, we also tested this association in the current study. In the analyses of global and subcortical brain morphometry, correction for multiple testing was performed using the Benjamini–Hochberg approach [49] for nine dietary patterns (four for age one year; five for age eight years) per five primary outcomes (45 tests in total) with a false discovery rate (FDR) of 0.05. In vertex-wise analyses of surface-based morphometry, the Gaussian Monte Carlo Simulations with a cluster-wise correction were used to correct for multiple testing. The cluster-forming threshold was set to *p* = 0.001, which corresponds to a false-positive rate of 0.05 [50]. Bonferroni corrections were further applied for each brain hemisphere (*p* < 0.025 cluster-wise).

Furthermore, we tested the mediating role of brain morphology in the associations of dietary pattern adherence with full scale IQ at age 13 years. To this end, we ran model-based causal mediation analyses for those dietary pattern—brain morphology associations that remained significant after multiple testing correction. For each mediation analysis, we specified mediator models for the conditional distribution of the diet-related brain morphology given the dietary pattern adherence and covariates, and outcome models for the conditional distribution of the full scale IQ given the dietary pattern adherence, diet-related brain morphology, and covariates. All models were adjusted for the same set of covariates in model 2. Each mediation analysis was ran with 1000 simulations using the quasi-Bayesian Monte Carlo method on normal approximation to obtain the estimates of the average direct, indirect, and total effects.

To reduce potential bias due to missing values on covariates, we applied multiple imputations by generating five independent datasets with 50 iterations using the MICE package in R. The mediation analyses were performed using ‘mediation’ package in R. The results of pooled analyses are presented. The analyses of surface-based morphometry were performed using QDECR package [51] in R version 3.6.3 and all other statistical analyses were carried out using R version 4.0.3 (R Foundation for Statistical Computing, Vienna, Austria). Two-sided *α* < 0.05 was considered statistical significance.

We performed additional analyses to test the robustness of the results. First, information on maternal and child characteristics between respondents and non-respondents with FFQ and neuroimaging data was compared. Second, we repeated multiple regression analyses restricted to children with a Dutch ethnic background, as the FFQs were developed and validated for Dutch children. Third, in addition to the primary aim of the study, which was associations of dietary patterns with global brain volumes, we also assessed if the observed associations for cerebral white matter and cerebral gray matter were present independent of a global effect (i.e., whether the difference observed in mean cerebral white matter and mean cerebral gray matter volume are proportionally different in relation to head size or the result of regional expansion of certain regions) by additionally adjusting for intracranial volume in model 2.

## Results

### Population characteristics

The characteristics of study participants is reported in Table 1. The majority of mothers was highly educated, had a high household income, and had a healthy lifestyle during pregnancy. The majority of children had a Dutch ethnic background. The information of missing values of covariates is listed in Supplemental Table 1.

**Table 1:** Characteristics of study population

	Study sample for dietary patterns at one year (N = 1888)	Study sample for dietary patterns at eight years (N = 2326)
Maternal characteristics at enrollment		
Educational level (Low), N (%)	548 (29.0%)	758 (32.6%)
*Household income per month, N (%)*		
< 1200 €	187 (9.9%)	221 (9.5%)
1200 – 2200 €	372 (19.7%)	512 (22.0%)
> 2200 €	1329 (70.4%)	1593 (68.5%)
*Alcohol use during pregnancy, N (%)*		
Never	640 (33.9%)	821 (35.3%)
Until pregnancy was known	274 (14.5%)	342 (14.7%)
Continued	974 (51.6%)	1163 (50.0%)
*Smoking during pregnancy, N (%)*		
Never	1484 (78.6%)	1840 (79.1%)
Until pregnancy was known	187 (9.9%)	212 (9.1%)
Continued	215 (11.4%)	274 (11.8%)
*Folic acid use, N (%)*		
No	255 (13.5%)	391 (16.8%)
Started the first 10 weeks	598 (31.7%)	730 (31.4%)
Started periconceptional	1035 (54.8%)	1205 (51.8%)
Psychopathological symptoms, median (IQR)	0.1 (0.1, 0.3)	0.1 (0.1, 0.3)
Diet quality scores during pregnancy, mean (SD)	7.9 (1.5)	7.8 (1.5)
*Child characteristics*		
Age at the neuroimaging assessment, median (IQR), years	9.9 (9.7, 10.0)	9.9 (9.7, 10.2)
Sex (Girls), N (%)	974 (51.6%)	1179 (50.7%)
*Ethnic background*		
Dutch	1346 (71.3%)	1568 (67.4%)
Non-Dutch Western	151 (8.0%)	223 (9.5%)
Non-Dutch Non-Western	393 (20.8%)	535 (23.0%)
Diet quality scores, mean (SD)	4.3 (1.4)	4.5 (1.2)

*IQR* interquartile range, *SD* standard deviation. Values are mean (SD) for continuous variables with a normal distribution, medians (IQR) for continuous variables with a skewed distribution, or valid numbers N (%) for categorical variables. Missing data of covariates (Supplemental Table 1) were imputed with multiple imputation (M = 5 imputations)

### Dietary patterns

The characteristics of diet quality scores and dietary patterns among children at one and eight years-of-age are shown in Table 2 and Table 3**,** respectively.

**Table 2:** Characteristics of the a priori*-*defined and a posteriori-derived dietary patterns, identified among children at age one year (N = 3629) ^1, 2^

			A posteriori-derived dietary patterns		
Food groups ^3^	Mean intake (g/d)	DQS-1y ^4^	Vegetables, potatoes and grains	Snacks, processed foods and sugar	Butter and margarines, whole grains and dairy
Vegetable	56	+	**0.79**	0.13	0.04
Potatoes	35	+	**0.69**	0.10	0.07
Pasta, rice and other grains	27	+	**0.59**	0.16	0.01
Vegetable oils	1	+	**0.53**	**0.35**	0.03
Composite meals	22	Not included	**0.45**	0.00	0.04
Meat	10	+	**0.41**	− 0.04	**0.26**
Fish and fish products	9	+	**0.40**	0.12	0.07
Children's meals	70	Not included	− **0.38**	− **0.23**	− 0.03
Legumes	5	+	**0.37**	**0.21**	− 0.05
Fruit	152	+	**0.27**	− **0.25**	**0.20**
Savory snacks	3	−	0.11	**0.61**	0.08
Refined cereals	17	Not included	− 0.01	**0.58**	0.14
Soups and bouillons	17	Not included	0.07	**0.52**	− **0.20**
Sugar and confectionery	29	−	0.01	**0.50**	**0.43**
Eggs	2	+	**0.20**	**0.44**	− 0.01
Sauces and condiments	2	Not included	**0.28**	**0.41**	0.15
Other fats	2	+	**0.24**	**0.36**	0.17
High-fat dairy products	52	+	0.05	**0.25**	0.09
Non-sugar-containing beverages	69	Not included	0.16	**0.22**	− 0.07
Nuts and seeds	1	Not included	0.07	0.18	0.17
Soy products	5	Not included	0.02	0.06	0.01
Butters and margarines	8	+	0.08	0.06	**0.60**
Whole grains	56	+	0.16	− **0.41**	**0.53**
Formula and breastfeeding	416	Not included	0.03	0.02	− **0.53**
Meat products	14	+	**0.27**	0.09	**0.52**
Low-fat dairy products	166	+	0.05	0.06	**0.51**
Sugar-containing beverages	194	−	− 0.07	**0.23**	**0.50**
Eigenvalues			4.0	1.9	1.6
Variance explained, % ^5^			10.9	9.1	8.1

DQS-1y, diet quality score at one year-of-age

^1^Factor loadings with and absolute value ≥ 0.20 are shown in bold

^2^Total sample with FFQ data at one year

^3^Included food items in the food groups are presented in Supplemental Table 2

^4^DQS-1y was quantified using ten food components with intake cut-offs based on age-specific dietary recommendations in the Dutch dietary guidelines. The signs indicate whether a food component was considered as an adequacy component ( +) or a moderation component (−) in the score

^5^Total explained variance by all three a posteriori-derived patterns is 28.1%

**Table 3:** Characteristics of the a priori*-*defined and a posteriori-derived dietary patterns, identified among children at age eight years (N = 4733)^1,2^

Food groups^3^	Mean intake (g/d)	DQS-8y^4^	A posteriori-derived dietary patterns			
			Snacks, potatoes and processed food	Fish, vegetables and fruit	Whole grains, soft fats and dairy	Meat replacement, legumes and nuts
Snacks and fast food	21	Not included	**0.61**	− 0.07	− 0.14	− 0.02
Potatoes	30	Not included	**0.60**	0.08	0.14	0.11
Sauces	5	Not included	**0.53**	− 0.06	− 0.04	− 0.04
Composite and ready-to-eat meals	66	Not included	**0.48**	0.14	**0.20**	0.15
Refined cereals and grains	47	Not included	**0.45**	0.14	− **0.39**	0.01
Processed meat	42	−	**0.45**	0.12	**0.25**	− **0.43**
Sugar and confectionery products	216	−	**0.43**	− 0.12	0.03	− 0.04
Sugar-containing beverages	186	−	**0.35**	− 0.03	− 0.15	0.01
Low- and moderate-fat fish	5	+	− 0.05	**0.61**	− 0.11	− 0.04
Vegetables	92	+	0.15	**0.55**	**0.34**	0.13
Fat fish	10	+	0.08	**0.50**	− 0.06	− 0.04
Fruit	121	+	− 0.14	**0.45**	**0.28**	0.02
Low-sugar-containing beverages	314	Not included	− 0.03	**0.41**	0.02	0.06
Hard fats	1	Not included	− 0.14	**0.35**	− 0.12	**0.25**
White meat, unprocessed	14	Not included	**0.24**	**0.32**	− 0.16	− **0.32**
Eggs	10	Not included	**0.21**	**0.23**	− 0.18	0.15
Whole grains	98	+	− 0.13	0.11	**0.76**	0.13
Soft fats	12	+	0.15	− 0.19	**0.62**	− 0.15
Low-fat dairy products	201	+	− 0.03	0.04	**0.35**	− **0.32**
Meat replacement products	4	Not included	− 0.05	0.03	0.19	**0.59**
Legumes	9	+	0.33	0.19	− 0.02	**0.37**
Red meat, unprocessed	10	Not included	**0.31**	**0.22**	0.00	− **0.37**
Nuts and nut butters	6	+	0.08	0.01	**0.20**	**0.36**
High-fat dairy products	35	+	0.05	0.15	− 0.07	**0.32**
Soy drinks	9	Not included	0.00	0.02	− 0.06	**0.23**
Porridge	2	Not included	0.04	0.00	− 0.09	0.14
Eigenvalues			2.5	1.9	1.7	1.4
Variance explained, % ^5^			9.2	7.0	6.9	5.8

*DQS-8y* diet quality score at eight years-of-age

^1^Factor loadings with and absolute value ≥ 0.20 are shown in bold

^2^Total sample with FFQ data at eight years

^3^Included food items in the food groups are presented in Supplemental Table 3

^4^DQS-8y was quantified using ten food components with intake cut-offs based on age-specific dietary recommendations in the Dutch dietary guidelines. The signs indicate whether a food component was considered as an adequacy component ( +) or a moderation component (−) in the score

^5^Total explained variance by all four a posteriori derived patterns is 28.8%

The mean DQS-1y was 4.3 (± 1.4). With PCA, three dietary patterns were identified, as shown in Table 2, which were named as: (1) ‘Vegetables, potatoes and grains’ dietary pattern; (2) ‘Snacks, processed foods and sugar’ dietary pattern; (3) ‘Butter and margarines, whole grains and dairy’ dietary pattern. Together, these three dietary patterns explained 28.1% of the variation in food intake of one-year-old children.

The mean DQS-8y was 4.5 (± 1.2). Four dietary patterns were identified by PCA, as shown in Table 3, which were named as: (1) ‘Snacks, potatoes and processed foods’ dietary pattern; (2) ‘Fish, vegetables and fruit’ dietary pattern; (3) ‘Whole grains, soft fats and dairy’ dietary pattern; (4) ‘Meat replacement, legumes and nuts’ dietary pattern. In total, the four dietary patterns explained 28.8% of the variation in food intake of eight-year-old children.

Correlations between a priori and a posteriori dietary patterns were calculated by the Pearson correlation coefficient and reported in Supplemental Table 4. DQS-1y is highly correlated with the ‘Vegetables, potatoes and grains’ dietary pattern at the same age (*r* = 0.69). DQS-8y is moderately associated with the ‘Whole grains, soft fats and dairy’ dietary pattern at age eight years (*r* = 0.56).

**Table 4:** Association of dietary patterns at ages one and eight years with global brain volumes at age 10 years

	Total brain volume, cm^3^			Cerebral white matter volume, cm^3^			Cerebral gray matter volume, cm^3^		
	*B*	95% CI	*p* value	*B*	95% CI	*p* value	*B*	95% CI	*p* value
*Diet at one year*									
Principal components									
Vegetables, potatoes and grains	− 2.2	− 6.8, 2.3	0.34	− 0.6	− 2.8, 1.5	0.56	− 1.4	− 3.8, 1.1	0.27
Snacks, processed foods and sugar	− **7.1**	− **12.8, **− **1.5**	**0.01**	− **4.3**	− **6.9, **− **1.7**	< 0.001*	− 2.5	− 5.5, 0.5	0.10
Butter and margarines, whole grains and dairy	4.4	− 0.2, 8.8	0.06	1.2	− 0.9, 3.3	0.25	**2.6**	**0.2, 4.9**	**0.03**
DQS-1y	1.5	− 2.7, 5.8	0.48	1.1	− 0.9, 3.1	0.27	0.3	− 2.0, 2.5	0.81
*Diet at eight years*									
Principal components									
Snacks, potatoes and processed foods	− **7.6**	− 13.2, − 2.1	**0.01**	− 2.5	− 5.1, 0.1	0.06	− **4.1**	− **7.0, **− **1.1**	**0.01**
Fish, vegetables and fruit	− 1.7	− 5.6, 2.3	0.40	− 0.1	− 2.0, 1.7	0.88	− 1.5	− 3.6, 0.6	0.16
Whole grains, soft fats and dairy	**8.9**	**4.5, 13.3**	< 0.001*	**3.0**	**0.9, 5.0**	**0.005**	**5.2**	**2.9, 7.5**	< 0.001*
Meat replacement, legumes and nuts	− 3.5	− 7.4, 0.4	0.08	− **1.8**	− **3.6, **− **0.03**	**0.046**	− 1.1	− 3.1, 1.0	0.30
DQS-8y	3.6	− 0.5, 7.7	0.08	1.3	− 0.6, 3.2	0.18	**2.3**	**0.2, 4.5**	**0.04**

*DQS-1y* diet quality score at one year-of-age, *DQS-8y* diet quality score at eight years-of-age, *CI* confidence interval. The effect estimates represent the difference in cubic centimeters for brain volumes per 1 SD higher score on the dietary pattern. This table present the results in model 2. Model 2 was adjusted for child sex, age when brain imaging was assessed, maternal education, household income, child ethnic background, child energy intake, child BMI measured at the age of 10 years, maternal diet quality during pregnancy, smoking during pregnancy, alcohol use during pregnancy, folic acid use, and maternal psychopathological symptoms during pregnancy. Statistical significance (*p* < 0.05) in model 2 is indicated with bold

^*^Denotes the associations which remained statistically significant after the Benjamini Hochberg correction for multiple testing (45 tests) with a FDR ≤ 0.05

### Associations of a priori and a posteriori dietary patterns with global and subcortical brain morphometry

Table 4 reports the associations of dietary patterns with global brain volumes, adjusted for covariates (model 2). A higher adherence to the ‘Snacks, processed foods and sugar’ dietary pattern at the age of one year was negatively associated with total brain (− 7.1 cm^3^/SD; 95% CI − 12.8, − 1.5) and cerebral white matter volumes (− 4.3 cm^3^/SD; 95% CI − 6.9, − 1.7) at age 10 years, while a higher adherence to the ‘Butter and margarines, cereals and dairy’ dietary pattern at age one year was positively associated with cerebral gray matter volume at age 10 years (2.6 cm^3^/SD; 95% CI: 0.2, 4.9). Only the association of cerebral white matter volume remained significant after correction for multiple testing. 

A higher adherence to the ‘Snacks, potatoes and processed foods’ dietary pattern at the age of eight years was negatively associated with total brain (− 7.6 cm^3^/SD; 95% CI − 13.2, − 2.1) and cerebral gray matter volumes (− 4.1 cm^3^/SD; 95% CI: − 7.0, − 1.1) at age 10. Conversely, a higher adherence to the ‘Whole grains, soft fats and dairy’ dietary pattern was positively associated with total brain (8.9 cm^3^/SD; 95% CI: 4.5, 13.3), cerebral white matter (3.0 cm^3^/SD; 95% CI: 0.9, 5.0) and cerebral gray matter volumes (5.2 cm^3^/SD; 95% CI: 2.9, 7.5). A higher DQS-8y was positively associated with cerebral gray matter volume (2.3 cm^3^/SD; 95% CI: 0.2, 4.5). The association of the ‘Whole grains, soft fats and dairy’ dietary pattern with total brain and cerebral gray matter volume remained present after multiple testing correction. The results of model 1 with minimal covariates adjustment are presented in Supplemental Table 5.

We observed no associations between dietary patterns at age one or eight years and hippocampal or amygdala volume at age 10 years (Supplemental Table 6), neither for left hippocampal volume (data not shown).

### Associations of a priori and a posteriori dietary patterns with surface-based brain morphometry

**Fig. 2:**
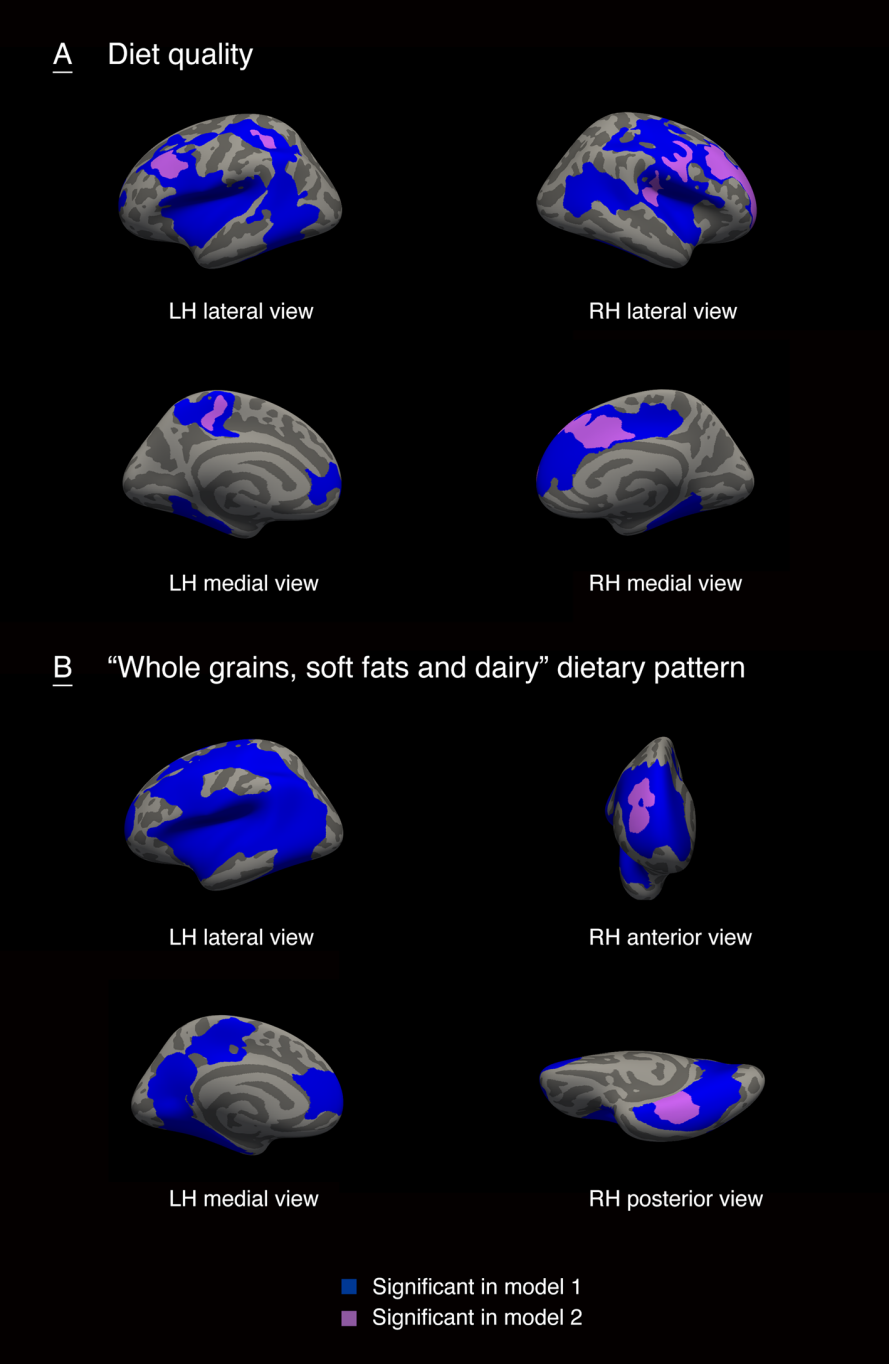
The associations of diet quality and a ‘Whole grains, soft fats and dairy’ dietary pattern at eight years-of-age with gyrification in children. LH, left hemisphere; RH, right hemisphere. Model 1 was adjusted for child sex and age when brain imaging was assessed. Model 2 was additionally adjusted for maternal education, household income, child ethnic background, maternal diet quality during pregnancy, smoking during pregnancy, alcohol use during pregnancy, folic acid use, maternal psychopathological symptoms, and BMI measured at the age of 10 years. Colored clusters represent regions of the brain that were positively associated with diet quality (**A**) or a ‘Whole grains, soft fats and dairy’ dietary pattern (**B**) that remained after the cluster-wise correction for multiple comparisons (*p* < 0.001)

**Fig. 3:**
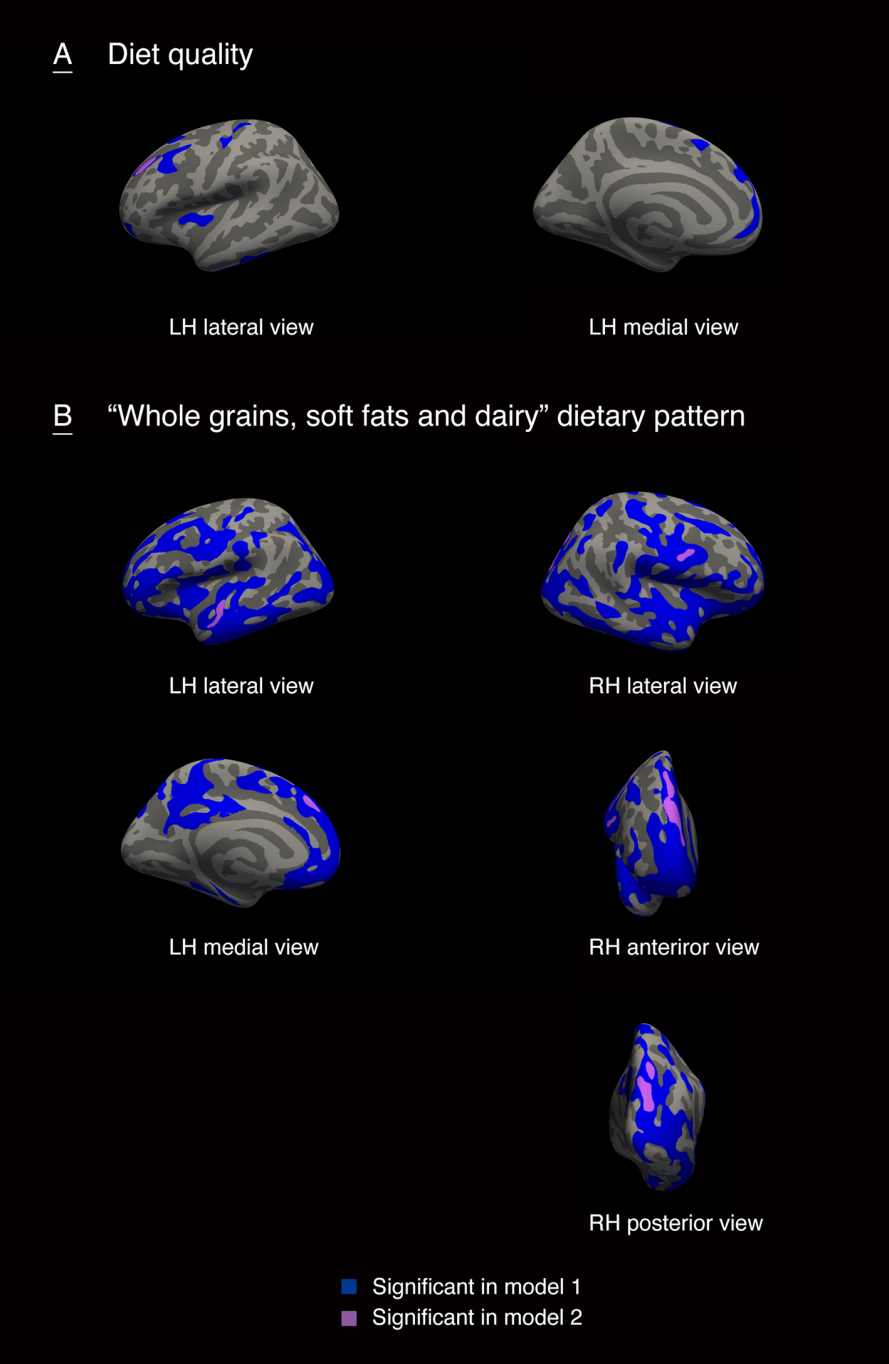
The associations of diet quality and a ‘Whole grains, soft fats and dairy’ dietary pattern at eight years-of-age with surface area in children. LH, left hemisphere; RH, right hemisphere. Model 1 was adjusted for child sex and age when brain imaging was assessed. Model 2 was additionally adjusted for maternal education, household income, child ethnic background, maternal diet quality during pregnancy, smoking during pregnancy, alcohol use during pregnancy, folic acid use, maternal psychopathological symptoms, and BMI measured at the age of 10 years. Colored clusters represent regions of the brain that were positively associated with diet quality (**A**) or a ‘Whole grains, soft fats and dairy’ dietary pattern (**B**) that remained after the cluster-wise correction for multiple comparisons (*p* < 0.001)

The cortical regions that were associate with DQS-8y and a ‘Whole grains, soft fats and dairy’ dietary pattern at eight years of age were illustrated in Figs. 2 and 3. We considered the regions identified as statistically significant in model 2 as the main results. Higher DQS-8y was positively associated with gyrification in regions of the frontal, parietal and temporal lobes, including rostral middle frontal gyrus in both hemisphere, postcentral and transverse temporal gyrus in the right hemisphere, and superior parietal and paracentral gyrus in the left hemisphere (Fig. 2A). A higher adherence to the ‘Whole grains, soft fats and dairy’ dietary pattern at age eight years was positively associated with gyrification in small regions of fusiform and rostral middle frontal gyrus in the right hemisphere (Fig. 2B). The positive association of higher DQS-8y with surface area was found in small clusters in rostral middle frontal region in the left hemisphere (Fig. 3A). Likewise, positive associations of a higher adherence to the ‘Whole grains, soft fats and dairy’ dietary pattern at age eight years with surface area were observed in small clusters in prefrontal and occipital lobe, including superior frontal region in both hemisphere, superior parietal and precentral region in the right hemisphere, and superior temporal in the left hemisphere (Fig. 3B). No associations were found between cortical thickness with DQS-8y or the ‘Whole grains, soft fats and dairy’ dietary pattern at eight years. In addition, we did not find significant associations of diet quality at the age of one year with either of the three surface-based cortical measures. Specific information on the associated anatomical regions and their cluster-wise *p* values are reported in the Supplemental Table 7.

### Mediation of dietary patterns and cognitive performance through diet-related brain morphology

**Fig. 4:**
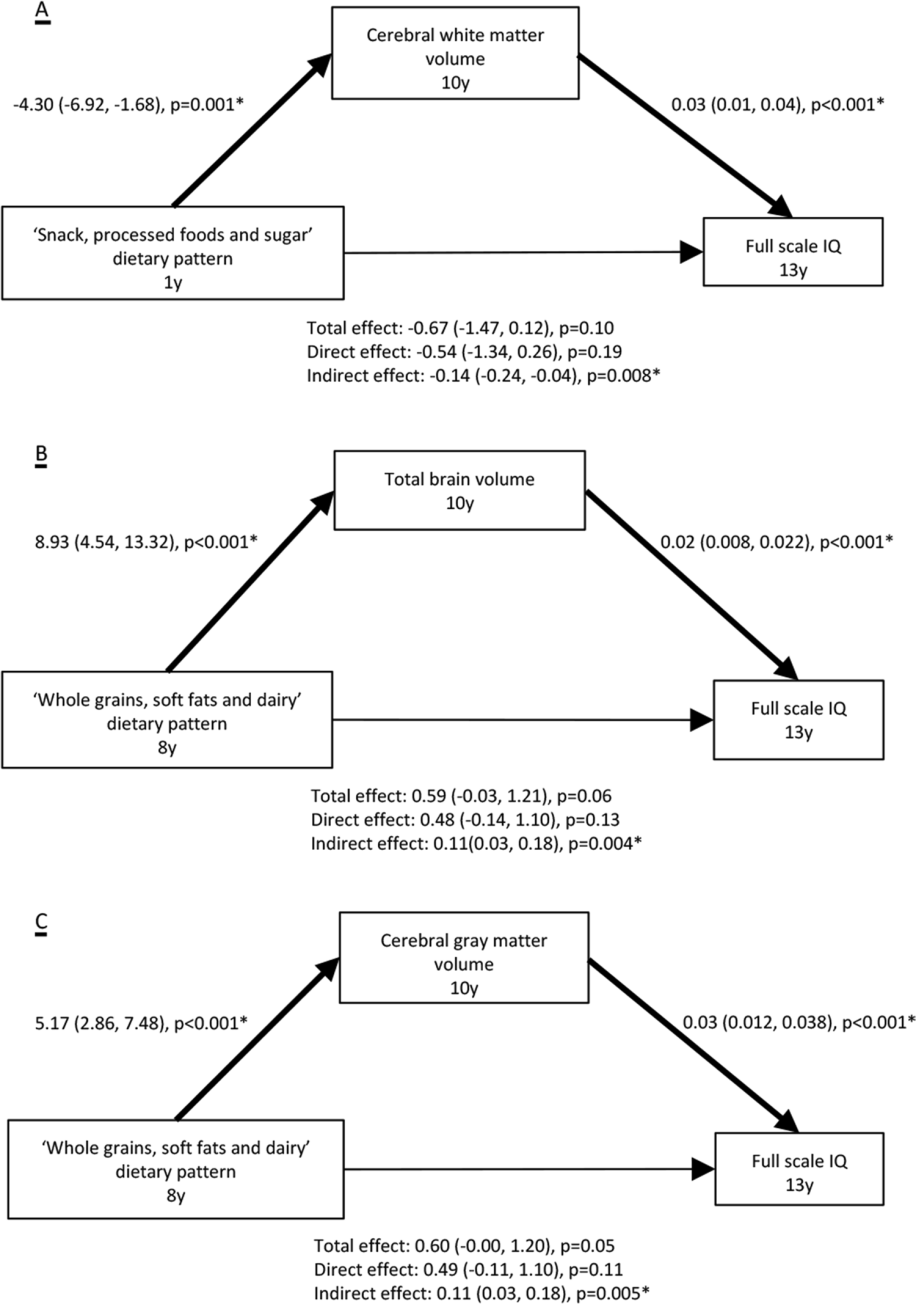
Global brain volumes mediate the association between dietary patterns adherence and full scale IQ at age 13 years. Estimates represent the standardized coefficients (95% CIs) for each pathway, adjusted for the child sex, age when brain imaging was assessed, maternal education, household income, child ethnic background, child energy intake, child BMI measured at the age of 10 years, maternal diet quality during pregnancy, smoking during pregnancy, alcohol use during pregnancy, folic acid use, and maternal psychopathological symptoms during pregnancy. *Denotes the estimate being statistically significant. **(A)** Total brain volume mediates the association between adherence to the ‘Whole grains, soft fats and dairy’ dietary pattern at age eight year and full scale IQ at age 13 years; **(B)** Total brain volume mediates the association between adherence to the ‘Whole grains, soft fats and dairy’ dietary pattern at age eight year and full scale IQ at age 13 years; **(C)** Cerebral gray matter volume mediates the association between adherence to the ‘Whole grains, soft fats and dairy’ dietary pattern at age eight year and full scale IQ at age 13 years

The mediation analyses were performed on dietary patterns that were significantly associated with global brain volumes and surface-based brain measures after multiple testing correction. The results, adjusting for all covariates, revealed that total brain, cerebral white and cerebral gray matter volumes mediated the associations of differences in adherence to dietary patterns at age one and eight years with full scale IQ at age 13 (Fig. 4)). As for surface-based measures, we found that clusters of significant gyrification and surface area mediated the relation between the diet quality at age eight years and IQ at age 13 (Supplemental Table 8), but not the ‘Whole grain, soft fats and dairy’ dietary pattern (data not shown).

### Additional analyses

First, the comparison of characteristics between respondents and non-respondents to FFQ and neuroimaging showed that respondents had a higher socioeconomic status, a higher percentage of folic acid usage before or during pregnancy, a better diet quality during pregnancy, and a higher percentage of children with a Dutch ethnic background (Supplemental Table 9). Second, the analyses including only children with a Dutch ethnic background showed stronger effect estimates and found more statistically significant associations in general, compared to the whole study population (Supplemental Table 10 and 11). Specifically, a higher adherence to the ‘Snacks, potatoes and processed foods’ at eight years was associated with decreased total brain and cerebral gray matter volume; a higher adherence to the ‘Whole grains, soft fats and dairy’ at eight years was associated with a larger total brain, cerebral white matter and cerebral gray matter volumes. While the effect size of association between the ‘Snacks, processed foods and sugar’ dietary pattern at age one year and cerebral white matter in the additional analysis was comparable to the main analysis, it was no longer significant. Third, when adding intracranial volume as a covariate in model 2, only the association between the category ‘Snacks, processed foods and sugar’ and cerebral white matter volume remained significant (− 1.4 cm^3^/SD; 95% CI − 2.8, − 0.04) after adjusting for multiple comparisons. This indicates that cerebral white matter volume may demonstrate regional volumetric expansion based on dietary patterns. In addition, we reported results without adjustment for diet quality during pregnancy in Supplemental Table 12. Comparing the results with the main results (model 2), the effect estimates were generally somewhat stronger. Furthermore, our results are robust when adjusting for breastfeeding duration and stressful life event in early childhood in addition to model 2 (data not shown).

## Discussion

This large population-based prospective cohort study explored associations of different dietary patterns in infancy and in mid-childhood with global, regional brain volumes and surface-based brain morphometry when children were 10 years of age. Diet quality at one and eight years-of-age as assessed by adherence to dietary guidelines was low to moderate. Global brain volumes at age 10 were negatively associated with dietary patterns characterized by high intake of snack and processed foods at age one and age eight years. Interestingly, global brain volumes were positively associated with a dietary pattern characterized by whole grains, soft fats and dairy at age eight years. No volumetric differences were found in the hippocampus or amygdala for dietary patterns at one and eight years-of-age. Higher DQS-8y or higher adherence to the aforementioned pattern with high intake in whole grains, soft fats and dairy at age eight years was associated with a greater gyrification and larger surface area in widespread areas of brain, with significant areas primarily clustered in association cortices, notably in the prefrontal cortex. Furthermore, global brain volumes, gyrification and surface area mediated the relationships between dietary patterns at ages one and eight years and children’s IQ at the age of 13 years. It is important to note that our study can only be interpreted in light of dietary patterns as a whole, but not on single food groups. In the following discussion, we compared the dietary patterns high in snacks and processed foods intake with western-like dietary patterns, while the dietary patterns high in whole grains intake were compared with prudent dietary patterns due to their high factor loadings for those food groups. Overall, our findings suggest that having a prudent dietary pattern in school age, specifically one rich in whole grains, soft fat and dairy, is linked to larger global brain volumes, whereas consuming a western-like dietary pattern in infancy and school age is associated with lower global brain volume. Our study provides novel information that extend earlier studies examining the association of human overall diet with brain health in childhood.

Our findings of the association between global brain morphology and children’s dietary patterns in early- and mid-childhood, especially those related to a prudent dietary pattern and a western-like dietary pattern, complement previous studies showing that overall dietary patterns are associated with school attainment [52, 53] and cognitive performance [15–17, 54, 55] in children. Considering that total brain volume [56], cerebral gray matter volume [57] and white matter microstructure [58] have been associated with cognitive ability of children, our results suggest that neuroanatomical correlates may underlie the association between dietary patterns and cognitive development in children. This hypothesis was further supported by the mediation analyses in this study, implying a mediating role of brain morphology in the association of dietary pattern and cognitive performance in children. Furthermore, our results indicate that a prudent or better diet quality was associated with brain regions functionally implicated in appetite regulation. Among those regions, most clustered in the dorsolateral prefrontal cortex (DLPFC). The prefrontal cortex is a complex region, but is notably involved in dopamine-mediated executive function, regulation of reward, and the inhibition of impulsive behaviors. Specifically, the DLPFC is associated with satiety, food craving, and executive functioning [59, 60]. A randomized controlled trail (RCT) in healthy young men with normal BMI found increased neuronal activity in right DLPFC reduced overall caloric intake and diminished self-reported appetite scores [61], which may suggest DLPFC involvement in food intake-related control mechanisms. However, it is unclear to what extent the functional connectivity translates into differences in brain morphology, although theories of gyrification support a relationship between folding patterns of the brain and increased connectivity [62]. Caution is needed regarding the translation of functional connectivity studies with the observed association between dietary patterns and brain morphology.

Among all significant associations, a prudent dietary pattern at age eight years was positively associated and a western-like dietary pattern at age one year was negatively associated with global brain volumes at age 10 years after multiple testing correction. The association of ‘Snacks, processed foods and sugar’ dietary pattern at one year with cerebral white matter volumes persisted after controlling for intracranial volume, suggesting that regional cerebral white matter development may be specifically susceptible to a western-like diet specifically in infancy. A possible mechanistic interpretation is that overall diet influences brain development if adherence to a dietary pattern with a higher risk of nutrient inadequacy occurs during a period of high need, considering the rapid rate of brain development within early life [1, 63]. The majority of white matter myelination occurs during the first 2 years of life [64]. Meanwhile, research has shown that children with a western-like dietary pattern were exposed to excess fat and sugar intake or inadequate nutrient intake, such as omega-3 polyunsaturated fatty acids [65], which serves as an important nutrient for nerve cell myelination and has shown beneficial effect on cognitive impairment [66, 67].

Contrary to the animal studies, our findings did not support our hypothesis that overall diet is associated with smaller hippocampal and amygdala volumes. We are aware of only one study investigating the effects of a western diet on hippocampal and amygdala volume in five- to nine-year-old-children. Stadterman et al. [30] reported a link between percentage of daily calories from fat, but not western diet, with an isolated decreased left hippocampal volume, but no relationship was found with the right hippocampal or amygdala volume. Their study calculated western diet as a summed percentage of daily calories from fat and sugar and the sample size was small (n = 21), however, it represents a promising step towards understanding the impact of western-like diet on hippocampus and amygdala development in children. We build upon their study investigating the association between a western-like diet and the left hippocampus but found no association. Our large sample size and the population-based sample, coupled with our approach to derive western-like dietary patterns, likely better resembles actual eating habits, although we did not observe evidence for any effects of either total or left hippocampal volume. Future research with longer follow-up time and in different study population is needed to ascertain the association of hippocampus and the amygdala with western-like diet in children.

Although the etiology of the relationships between dietary patterns and neurodevelopment remains unclear, there have been potential mechanisms proposed. First, the effect of dietary patterns on brain morphology may be mediated by epigenetic mechanisms. Diet, as an epigenetic programming regulator, affects multiple genes expression at levels of transcription, translation and post-translational modification. Subsequently, the variation in gene expression directly regulated by nutrition influences several neurobiological processes, including neurogenesis, synaptic plasticity and neuronal connectivity [68, 69], which may lead to rearrangement of brain structure. The epigenetic mechanism can be supported by a study using data from the Barbados Nutrition Study, which followed a group of adults who had been hospitalized during their infancy for protein-energy malnutrition. Compared with a control group of adults who were not exposed to malnutrition in their childhood, the researchers identified 134 nutrition-sensitive, differentially methylated genomic regions using epigenome-wide analysis, and found that methylation at some of these sites were associated with cognitive outcomes (i.e., IQ and attention) [70]. Second, differences in dietary patterns can induce differences in metabolic changes underlying brain morphological development. For example, brain-derived neurotrophic factor (BDNF), which plays a pivotal role in glucose and energy metabolism, is related to the morphological variation of the hippocampus and prefrontal cortex [71], and can be influenced by diet. High-fat diets have been found to increase oxidative stress, inflammation and further interfere with the level of BDNF [72]. Regarding healthy dietary patterns, a randomized control trial study in adults found a higher plasma BDNF levels in an experimental group with a Mediterranean diet intervention compared to a control group [73]. Furthermore, decreased BDNF has been linked to cognitive decline [72]. While these studies were performed in adults, there may be expectations of even greater differences in children, considering the high energy consumption associated with neurodevelopment. Further, the evidence showing dietary patterns were associated with cognitive performance in children, suggests shared brain metabolic pathways that underlie brain morphological changes in children and adults. Last, diet could affect the gut microbiota which in return alter the host’s physiological responses and associated structural adaption. Short-chain fatty acids (SCFAs) are the most studied metabolites produced by microbes. Foods high in dietary fiber, such as fruits, vegetables and whole grains, increase the levels of SCFAs through gut microbial fermentation [74]. Results from animal studies indicate that SCFAs might influence gut-brain communication and brain function directly or indirectly through neurochemical pathways [75].

By reporting associations of dietary patterns in early and mid-childhood with global and regional brain volumes, our findings underline the need for nutrition research related to child brain development, notably in middle childhood. Nutrition has been hypothesized as an aspect of the experience-dependent environment that can influence neurodevelopment [76]. However, most studies on nutrition and brain development have focused on early life nutrition, while few studies have examined children in the ‘forgotten years’. In fact, dietary patterns change drastically from milk-based during infancy to omnivore patterns in childhood. Previous work from our group showed that diet quality at one year-of-age changes considerably by eight years-of-age [12], indicating that relative stability of dietary patterns may occur after the age of one year. Another longitudinal study including five European countries suggests that dietary patterns are established between one and two years and remain stable to eight years of age [8]. In addition, brain metabolism associated with neurodevelopment is high, with a considerable need of energy. This changes at around the age of four to five years, coincident with the slower rate of growth during that age. Overall, changes in dietary patterns across childhood, highlight the importance of investigating the effect of diet not only in infancy, but also in mid-childhood.

Most dietary interventions for brain development to date have targeted micronutrient supplementation, showing positive effects of nutritional supplementation in certain cognitive domains in nutrient-deficient children [77]. Some interventions which used food supplementation (e.g., fortified foods) in early childhood [1] or in children with atypical neurodevelopment [78] have shown short-term effects on cognitive and motor development. Evidence from those RCTs is intriguing and denotes a potential causal link between nutrition and brain development. However, some RCT studies that provide supplements of a single nutrient or food in certain groups of children failed to detect changes in cognitive performance [79, 80]. It is noteworthy that none of RCTs considers overall diet at the baseline in their study design, which may partly explain the inconsistency in findings. This could be explained by an adapted hypothetical scenario in which the effects of nutrient adequacy and overall diet quality may show an interacting effect on children’s cognitive development [1], thus reducing the effect of nutrient supplementation. Moreover, it is largely unknown whether the effect of nutrition on brain development could be detected by objective anatomical measurement, like brain volumetric alteration. We filled in the gap of lack of evidence in a healthy pediatric population with a long follow-up period.

The strengths of our study are the population-based prospective design, availability of multiple covariates, and large-scale neuroimaging in children. In addition, we defined dietary patterns using a priori diet quality score and a posteriori dietary patterns based on food groups, which captured unrelated eating patterns in the population. Therefore, the results of our study provide a public health message on the relationships of dietary patterns and brain development in children.

The main limitation is the design of the study. Although longitudinal, the design is in essence cross-sectional, as we lack repeated measures of diet and brain imaging. Thus, our results cannot infer causality between dietary patterns and brain morphometry. Future studies with repeated measurements of dietary patterns and brain morphology, preferably those with an intervention component, are necessary to better understand the direction of the association. Another limitation involves assumptions associated with calculating the a priori and a posteriori dietary patterns analysis, which can influence the interpretation and limit the comparability of the study with others. Such assumptions include how food items were clustered into food groups, the number of principal components to be retained. Additionally, dietary patterns are likely to be a part of lifestyle behaviors, and thus there is the potential for residual confounding. However, after adjusting for BMI at the age of 10 years in the model, dietary patterns were still significantly associated with brain morphology. Several other limitations should also be acknowledged. First, the use of FFQs to quantify dietary intake is subject to measurement error [81]. However, the FFQs used in our study were validated against three 24 h recalls in Dutch children at age one year and against the doubly labelled water method in Dutch children at age eight years, and had sufficient capacity of ranking participants with regard to energy intake. Moreover, we adjusted our models for energy intake to mitigate the effect of measurement error in the FFQs that individuals tend to misreport their intake of food and beverages in the same direction. Fourth, although we adjusted for several covariates including socioeconomic status, maternal psychopathological symptoms, lifestyle and child BMI, we cannot rule out genetic and unmeasured environmental confounding because of the observational nature of the study. Lastly, the non-response analyses suggested a possible selection bias, which may limit the generalizability of the study.

## Conclusion

We found a relationship between early-life and mid-childhood dietary patterns and later brain morphology, and brain morphology mediated the association of dietary patterns with cognitive performance using a prospective population-based study of child development, which indicates that a healthy diet may benefit neurodevelopment, which has an important public health message. The findings of our study suggest that a prudent dietary pattern in mid-childhood is positively associated with global and widespread differences in pre-adolescent brain morphometry. Adherence to a western-like dietary pattern in infancy and school age is differentially associated with cerebral white matter, cerebral gray matter and total brain volume, suggesting that the timing of neurodevelopmental processes may play an important role in understanding the effect of overall diet. Furthermore, most diet-related brain morphological measures mediated the association of dietary patterns in childhood with full scale IQ in adolescence. With the observed temporal associations and mediation effect, our findings highlight the potential of overall dietary patterns to affect brain morphology in children, and that these differences in brain morphology may explain part of the previously demonstrated relation between dietary patterns and neurodevelopment and cognitive performance in children. However, further research with repeated measurements of dietary patterns and brain morphology is needed to better understand the direction of the association.

## Supplementary Information

Below is the link to the electronic supplementary material.

Supplementary file 1 (DOCX 228 KB)
