# Supplementary material for: Dietary patterns, brain morphology and cognitive performance in children: Results from a prospective population-based study
Source: Eur J Epidemiol. 2023 May 8;38(6):669–87. doi: 10.1007/s10654-023-01012-5 (PMC10232626; doi:10.1007/s10654-023-01012-5)
Supplement: Supplementary file 1 — (DOCX 228 KB) [file 10654_2023_1012_MOESM1_ESM.docx]

**Dietary patterns, brain morphology and cognitive performance in children: Results from a prospective population-based study**

Yuchan Mou^1,2^, Elisabet Blok^2, 3^, Monica Barroso^1^, Pauline W. Jansen^3, 4^, Tonya White^3, 5, 6^, Trudy Voortman^1^

^1^ Department of Epidemiology, Erasmus MC, University Medical Center, Rotterdam, the Netherlands;

^2^ The Generation R Study Group, Erasmus MC, University Medical Center, Rotterdam, the Netherlands;

^3^ Department of Child and Adolescent Psychiatry/Psychology, Erasmus MC, University Medical Center, Rotterdam, the Netherlands;

^4^ Department of Psychology, Education and Child Studies, Erasmus University Rotterdam, Rotterdam, the Netherlands;

^5^ Department of Radiology and Nuclear Medicine, Erasmus MC, University Medical Center, Rotterdam, the Netherlands;

^6^ Section on Social and Cognitive Developmental Neuroscience, National Institutes of Mental Health, Bethesda, Maryland, USA

**Corresponding author**: Trudy Voortman, [trudy.voortman@erasmusmc.nl](mailto:trudy.voortman@erasmusmc.nl)

Supplemental Table 1 Numbers and percentages of missing values of covariates before multiple imputation

|  | Study sample for dietary patterns at age one year (N = 1888) |  | Study sample for dietary patterns at age eight years (N = 2326) |
| --- | --- | --- | --- |
|  | Missing values N (%) | | |
| *Maternal characteristics* |  |  |  |
| Educational level | 154 (8.2) |  | 240 (10.3) |
| Household income per month | 247 (13.1) |  | 413 (17.8) |
| Alcohol use during pregnancy | 220 (11.7) |  | 352 (15.1) |
| Smoking during pregnancy | 159 (8.4) |  | 232 (10.0) |
| Folic acid use | 470 (24.9) |  | 643 (27.6) |
| Psychological symptoms | 328 (17.4) |  | 474 (20.4) |
| Diet quality scores during pregnancy | 435 (23.0) |  | 584 (25.1) |
|  |  |  |  |
| *Child characteristics* |  |  |  |
| Age at the neuroimaging assessment | - |  | - |
| Sex | - |  | - |
| Ethnic background | 6 (0.3) |  | 7 (0.3) |
| BMI at 10 years | 4 (0.2) |  | 4 (0.2) |

Supplemental Table 2 List of 27 food groups and corresponding food items, as considered in *a posteriori*-defined dietary pattern analyses at the age of one year ^1^

| **Food group** | **Included food items** |
| --- | --- |
| Refined cereals | Waffles, rusk crackers, currant bread, currant bun, white bread or baguette, croissant, cornflakes, low-fiber breakfast cereals |
| Whole cereals | Brown or whole-bran bread, brown or whole-bran baguette, oatmeal, muesli, multigrain breakfast cereals |
| Pasta, rice and other grains | Pasta, rice, couscous, bulgur |
| High-fat dairy products | Whole milk and flavored milk, whole yoghurt, *fromage frais* and custard, chocolate-flavored milk, milk pudding, mousse |
| Low-fat dairy products | Semi-skimmed or skimmed milk and flavored milk, semi-skimmed or skimmed yoghurt, *fromage frais* and custard, yoghurt drinks, porridge |
| Formula and breastfeeding | Infant milk formula and breastfeeding |
| Fruit | Fruit and fruit compotes (excluding fruit juice) |
| Soy products | Soy milk, soy flavored milk, soy dessert, soy-based meat substitutes |
| Vegetables | Raw, cooked and baked vegetables |
| Potatoes | Potatoes (including home-fried and baked potatoes) |
| Soups and bouillons | Soups and bouillons |
| Nuts and seeds | Nut and seed butters |
| Sauces and condiments | Mayonnaise (including half-fat), salad cream, peanut sauce, ketchup, and other sauces added to meals or snacks |
| Savory snacks | Chips, toasts with cheese or pâté, sausage rolls, spring rolls, meat rolls, meat croquettes, satay, peanuts and nuts, burgers, chicken nuggets, fried potatoes and chips |
| Sugar and confectionery | Dutch spiced honey cake, chocolate pasta, chocolate sprinkles, sweet sandwich fillings, cookies, biscuits, cakes, pastry, pancakes, added sugar, chocolate, candy, ice cream |
| Vegetable oils | Olive oil and other vegetable oils |
| Butters and margarines | Butter, full- and low-fat margarines |
| Other fats | Cooking, frying, and baking fats |
| Fish and fish products | Fish and fish products, shellfish |
| Meat | Non-processed meat |
| Meat products | Processed meat and meat products, excluding meat-containing snacks, which are included in “savory snacks” |
| Eggs | Eggs (baked or boiled) |
| Legumes | Legumes (white and brown beans, kidney beans, lentils, chickpeas) |
| Sugar-containing beverages | Softdrinks, fruit drinks, cordial |
| Non-sugar-containing beverages | Tea without sugar, water, diet soft drinks (without sugar) |
| Children's meals | Ready-to-eat infant meals and food pots |
| Composite meals | Composite dishes |

^1^ Table adapted from Monica Barroso et al., Gastroenterology, 2018.

Supplemental Table 3 List of 26 food groups and corresponding food items, as considered in *a posteriori*-defined dietary pattern analyses at the age of eight years

| **Food group** | **Included food items** |
| --- | --- |
| Fruit | Fresh fruit |
| Vegetables | Cooked and raw vegetables |
| Potatoes | Potatoes except fried and chips |
| Whole grains | Whole bread, grains and grain products |
| Refined cereals and grains | White bread, grains and grain products |
| Lean fish | Low and moderate fat fish, shellfish |
| Fatty fish | High-fat, canned and breaded fish |
| Red meat unprocessed | Unprocessed low-fat and high fat red meat |
| White meat unprocessed | Unprocessed white meat |
| Processed meat | Red and white processed meats |
| Meat replacement products | Meat replacement products |
| Soy drinks | Soy drinks |
| Legumes | Canned Legumes |
| Eggs | Eggs |
| Low fat dairy | Low-fat milk, yoghurt and cheese |
| High fat dairy | High-fat milk, yoghurt and cheese |
| Porridge | Porridge |
| Nuts and seeds | Nuts, seeds and nut butters |
| Hard fats | Hard fats |
| Soft fats | Soft fats (low SFA) |
| Sugary drinks | Soft drinks, lemonade and concentrate with sugar, fruit juices |
| Low-sugar drinks | Low-sugar soft drinks, lemonades and concentrates, tea |
| Sugar and confectionary products | Honey, sugar syrup, sweet toppings, sweet snacks, dried fruit, dairy desserts, milk beverages with sugar, yoghurt with sugar |
| Sauces | High-fat sauces and dressings |
| Snacks and fast food | Savory snacks, potato crisps, french fries, sausage rolls, meat-based fast food |
| Composite meals | Pizzas and savory pies, pasta dishes, Indonesian dishes, restaurant foods |

Supplemental Table 4 Correlations between *a priori*-defined and *a posteriori*-derived dietary patterns at one and eight years-of-age

| Diet at one year |  |  |  |  |
| --- | --- | --- | --- | --- |
|  | Vegetables, potatoes and grains | Snacks, processed foods and sugar | Butter and margarines, cereals, and dairy |  |
| DQS-1y | 0.69 | -0.18 | 0.33 |  |
| Diet at eight years |  |  |  |  |
|  | Snack, potatoes, and processed foods | Vegetables, fish and fruit | Whole grains, soft fats and dairy | Meat replacement, legumes and nuts |
| DQS-8y | 0.01 | 0.40 | 0.56 | 0.19 |

Abbreviations: DQS-1y, diet quality score at one year-of-age; DQS-8y, diet quality score at eight year-of-age.

Supplemental Table 5 Association of dietary patterns at ages one and eight years with global brain volumes at age 10 years

|  |  | Model 1 |  |  | Model 2 |  |
| --- | --- | --- | --- | --- | --- | --- |
|  | *B* | 95% CI | *p* value | *B* | 95% CI | *p* value |
| **Total brain volume (cm^3^)** |  |  |  |  |  |  |
| *Diet at one year* |  |  |  |  |  |  |
| Principal components |  |  |  |  |  |  |
| Vegetables, potatoes and grains | 0.24 | -4.23, 4.71 | 0.9164 | -2.24 | -6.81, 2.33 | 0.3358 |
| Snacks, processed foods and sugar | -17.03 | -21.85, -12.22 | <0.0001 | **-7.14** | **-12.79, -1.49** | **0.0134** |
| Butter and margarines, whole grains and dairy | 10.37 | 5.92, 14.81 | <0.0001 | 4.35 | -0.15, 8.84 | 0.0580 |
| DQS-1y | 7.26 | 3.04, 11.47 | 0.0007 | 1.52 | -2.73, 5.77 | 0.4836 |
|  |  |  |  |  |  |  |
| *Diet at eight years* |  |  |  |  |  |  |
| Principal components |  |  |  |  |  |  |
| Snacks, potatoes and processed foods | -11.62 | -15.46, -7.77 | <0.0001 | **-7.63** | **-13.19, -2.06** | **0.0073** |
| Fish, vegetables and fruit | 0.20 | -3.67, 4.07 | 0.9187 | -1.67 | -5.61, 2.27 | 0.4048 |
| Whole grains, soft fats and dairy | 18.13 | 14.29, 21.97 | <0.0001 | **8.93** | **4.54, 13.32** | **0.0001^*^** |
| Meat replacement, legumes and nuts | -3.74 | -7.68, 0.21 | 0.0634 | -3.51 | -7.38, 0.37 | 0.0764 |
| DQS-8y | 8.75 | 4.97, 12.53 | <0.0001 | 3.64 | -0.45, 7.74 | 0.0812 |
|  |  |  |  |  |  |  |
| **Cerebral white matter volume (cm^3^)** |  |  |  |  |  |  |
| *Diet at one year* |  |  |  |  |  |  |
| Principal components |  |  |  |  |  |  |
| Vegetables, potatoes and grains | 0.37 | -1.67, 2.4 | 0.7232 | -0.64 | -2.76, 1.48 | 0.5557 |
| Snacks, processed foods and sugar | -7.03 | -9.22, -4.83 | <0.0001 | **-4.30** | **-6.92, -1.68** | **0.0013^*^** |
| Butter and margarines, whole grains and dairy | 3.41 | 1.38, 5.44 | 0.001 | 1.22 | -0.86, 3.31 | 0.2501 |
| DQS-1y | 3.18 | 1.26, 5.09 | 0.0012 | 1.10 | -0.87, 3.07 | 0.2743 |
|  |  |  |  |  |  |  |
| *Diet at eight years* |  |  |  |  |  |  |
| Principal components |  |  |  |  |  |  |
| Snacks, potatoes and processed foods | -4.15 | -5.9, -2.4 | <0.0001 | -2.53 | -5.11, 0.06 | 0.0553 |
| Fish, vegetables and fruit | 0.47 | -1.28, 2.23 | 0.5979 | -0.14 | -1.97, 1.69 | 0.8800 |
| Whole grains, soft fats and dairy | 6.02 | 4.26, 7.77 | <0.0001 | **2.95** | **0.91, 4.99** | **0.0046** |
| Meat replacement, legumes and nuts | -1.85 | -3.64, -0.06 | 0.0427 | **-1.83** | **-3.63, -0.03** | **0.0463** |
| DQS-8y | 3.02 | 1.3, 4.74 | 0.0006 | 1.32 | -0.59, 3.22 | 0.1754 |
|  |  |  |  |  |  |  |
| **Cerebral gray matter volume (cm^3^)** |  |  |  |  |  |  |
| *Diet at one year* |  |  |  |  |  |  |
| Principal components |  |  |  |  |  |  |
| Vegetables, potatoes and grains | -0.1 | -2.46, 2.26 | 0.9351 | -1.35 | -3.76, 1.06 | 0.2706 |
| Snacks, processed foods and sugar | -8.52 | -11.06, -5.97 | <0.0001 | -2.52 | -5.50, 0.46 | 0.0974 |
| Butter and margarines, whole grains and dairy | 5.83 | 3.48, 8.17 | <0.0001 | **2.57** | **0.20, 4.94** | **0.0334** |
| DQS-1y | 3.36 | 1.14, 5.59 | 0.0031 | 0.27 | -1.96, 2.51 | 0.8113 |
|  |  |  |  |  |  |  |
| *Diet at eight years* |  |  |  |  |  |  |
| Principal components |  |  |  |  |  |  |
| Snacks, potatoes and processed foods | -6.05 | -8.08, -4.02 | <0.0001 | **-4.07** | **-7.00, -1.14** | **0.0065** |
| Fish, vegetables and fruit | -0.38 | -2.42, 1.67 | 0.7164 | -1.50 | -3.58, 0.58 | 0.1569 |
| Whole grains, soft fats and dairy | 10.34 | 8.32, 12.36 | <0.0001 | **5.17** | **2.86, 7.48** | **<0.0001^*^** |
| Meat replacement, legumes and nuts | -1.25 | -3.33, 0.84 | 0.2404 | -1.07 | -3.11, 0.97 | 0.3040 |
| DQS-8y | 5.19 | 3.19, 7.19 | <0.0001 | **2.30** | **0.15, 4.45** | **0.0358** |

Abbreviations: DQS-1y, diet quality score at one year-of-age; DQS-8y, diet quality score at eight year-of-age; CI confidence interval. The effect estimates represent the difference in cubic centimeters for brain volumes per 1 SD higher score on the dietary pattern. This table present the results in Model 1. Model 1 was adjusted for child sex and age when brain imaging was assessed.

Supplemental Table 6 Association of dietary patterns at ages one and eight years with regional brain volumes at age 10 years

|  |  | Model 1 |  |  |  | Model 2 |  |
| --- | --- | --- | --- | --- | --- | --- | --- |
|  | *B* | 95% CI | *p* value |  | *B* | 95% CI | *p* value |
| **Hippocampal volume (mm^3^)** |  |  |  |  |  |  |  |
| *Diet at one year* |  |  |  |  |  |  |  |
| Principal components |  |  |  |  |  |  |  |
| Vegetables, potatoes and grains | 19.86 | -11.86, 51.58 | 0.2196 |  | 10.27 | -18.63, 39.17 | 0.4861 |
| Snacks, processed foods and sugar | -63.86 | -98.36, -29.37 | 0.0003 |  | 6.82 | -29.15, 42.79 | 0.7100 |
| Butter and margarines, whole grains and dairy | 59.37 | 27.77, 90.97 | 0.0002 |  | 21.55 | -6.92, 50.02 | 0.1378 |
| DQS-1y | 46.69 | 16.78, 76.6 | 0.0022 |  | 9.75 | -17.16, 36.66 | 0.4774 |
|  |  |  |  |  |  |  |  |
| *Diet at eight years* |  |  |  |  |  |  |  |
| Principal components |  |  |  |  |  |  |  |
| Snacks, potatoes and processed foods | -56.85 | -85.27, -28.44 | 0.0001 |  | -14.82 | -51.65, 22 | 0.4298 |
| Fish, vegetables and fruit | 3.92 | -24.58, 32.42 | 0.7875 |  | -10.46 | -36.29, 15.37 | 0.4271 |
| Whole grains, soft fats and dairy | 69.85 | 41.25, 98.46 | <0.0001 |  | -17.52 | -46.61, 11.58 | 0.2378 |
| Meat replacement, legumes and nuts | -2.66 | -31.73, 26.41 | 0.8575 |  | 14.17 | -11.3, 39.63 | 0.2755 |
| DQS-8y | 27.6 | -0.36, 55.56 | 0.0530 |  | -27.24 | -54.48, 0.00 | 0.0500 |
|  |  |  |  |  |  |  |  |
| **Amygdala volume (mm^3^)** |  |  |  |  |  |  |  |
| *Diet at one year* |  |  |  |  |  |  |  |
| Principal components |  |  |  |  |  |  |  |
| Vegetables, potatoes, and grains | 12.13 | -4.62, 28.88 | 0.1558 |  | 4.20 | -11.08, 19.48 | 0.5897 |
| Snacks, processed foods and sugar | -34.01 | -52.23, -15.79 | 0.0003 |  | 0.68 | -18.12, 19.48 | 0.9435 |
| Butter and margarines, whole grains and dairy | 33.1 | 16.41, 49.78 | 0.0001 |  | 7.69 | -7.33, 22.7 | 0.3154 |
| DQS-1y | 29.6 | 13.82, 45.38 | 0.0002 |  | 8.04 | -6.15, 22.22 | 0.2666 |
|  |  |  |  |  |  |  |  |
| *Diet at eight years* |  |  |  |  |  |  |  |
| Principal components |  |  |  |  |  |  |  |
| Snacks, potatoes and processed foods | -27.37 | -41.74, -13.01 | 0.0002 |  | -11.21 | -29.65, 7.23 | 0.2334 |
| Fish, vegetables and fruit | 2.96 | -11.45, 17.36 | 0.6871 |  | -4.93 | -17.99, 8.13 | 0.4592 |
| Whole grains, soft fats and dairy | 43.46 | 29.04, 57.89 | <0.0001 |  | 2.11 | -12.58, 16.79 | 0.7785 |
| Meat replacement, legumes and nuts | -9.25 | -23.94, 5.44 | 0.2170 |  | 1.07 | -11.85, 13.98 | 0.8712 |
| DQS-8y | 16.92 | 2.79, 31.04 | 0.0189 |  | -8.49 | -22.09, 5.11 | 0.2210 |

Abbreviations: DQS-1y, diet quality score at one year-of-age; DQS-8y, diet quality score at eight year-of-age; CI confidence interval. The effect estimates represent the difference in cubic centimeters for brain volumes per 1 SD higher score on the dietary pattern. Model 1 was adjusted for child sex and age when brain imaging was assessed. Model 2 was additionally adjusted for maternal education, household income, child ethnic background, child energy intake, child BMI measured at the age of 10 years, maternal diet quality during pregnancy, smoking during pregnancy, alcohol use during pregnancy, folic acid use, maternal psychopathological symptoms during pregnancy and child intracranial volume.

Supplemental Table 7 Brain regions that are associated with dietary patterns at age eight years and the corresponding *p* values

| Cortical surface measures | Hemisphere | Anatomical Region | Area Size (mm^2^) | Coordinates | | | Mean coefficient | Cluster-wise *p* value ^1^ |
| --- | --- | --- | --- | --- | --- | --- | --- | --- |
|  |  |  |  | x | y | z |  |  |
| DQS-8y | | | | | | | | |
| Gyrification | RH | Rostral middle frontal | 6319.89 | 24.8 | 49.6 | 6.3 | 0.02 | 0.0001 |
|  |  | Postcentral | 870.25 | 52.0 | -7.4 | 21.7 | 0.02 | 0.0001 |
|  |  | Transverse temporal | 723.66 | 45.7 | -25.2 | 6.9 | 0.03 | 0.0002 |
|  | LH | Rostral middle frontal | 901.65 | -41.8 | 25.2 | 36.1 | 0.02 | 0.0001 |
|  |  | Superior parietal | 416.50 | -34.2 | -43.6 | 42.4 | 0.02 | 0.0123 |
|  |  | Paracentral | 350.10 | -6.5 | -35.0 | 54.1 | 0.01 | 0.0243 |
| Surface area | RH | - | - | - | - | - | - | - |
|  | LH | Rostral middle frontal | 259.34 | -22.9 | 37.0 | 32.7 | 0.04 | 0.0013 |
| “Whole grains, soft fats and dairy” dietary pattern at eight years | | | | | | | | |
| Gyrification | RH | Fusiform | 1212.01 | 37.6 | -29.1 | -25.4 | 0.02 | 0.0001 |
|  |  | Rostral middle frontal | 1076.00 | 25.8 | 40.0 | 27.7 | 0.02 | 0.0001 |
|  | LH | - | - | - | - | - | - | - |
| Surface area | RH | Superior frontal | 699.33 | 9.3 | 51.0 | 26.8 | 0.02 | 0.0001 |
|  |  | Superior parietal | 438.70 | 19.6 | -87.0 | 30.9 | 0.02 | 0.0001 |
|  |  | Superior parietal | 156.15 | 21.1 | -69.9 | 41.3 | 0.02 | 0.0192 |
|  |  | Precentral | 151.18 | 58.8 | 2.6 | 27.7 | 0.02 | 0.0221 |
|  | LH | Superior temporal | 250.92 | -58.8 | -8.2 | -3.8 | 0.02 | 0.0012 |
|  |  | Superior frontal | 165.30 | -8.2 | 30.4 | 44.3 | 0.01 | 0.0149 |

Abbreviations: DQS-8y, diet quality score at eight year-of-age; RH, right hemisphere; LH, left hemisphere. The models were adjusted for child sex and age when brain imaging was assessed, maternal education, household income, child ethnic background, child energy intake, child BMI measured at the age of 10 years, maternal diet quality during pregnancy, smoking during pregnancy, alcohol use during pregnancy, folic acid use, and maternal psychopathological symptoms during pregnancy.

^1^ Cluster-wise *p* values were derived from vertex-wise analyses using the Gaussian Monte Carlo Simulations at the cluster level. The cluster-forming threshold was *p* = 0.001. Bonferroni corrections were further applied for each brain hemisphere, which corresponding to *p* < 0.025.

Supplemental Table 8 Clusters of significant gyrification and surface area mediates the association between DQS-8y and full scale IQ at age 13 years (N=2326)

|  |  | Total effect  (95% CI) | *p* value | Direct effect  (95% CI) | *p* value | Indirect effect  (95% CI) | *p* value |
| --- | --- | --- | --- | --- | --- | --- | --- |
| DQS-8y | | | | | | | |
| Gyrification |  |  |  |  |  |  |  |
| RH | Rostral middle frontal | 0.81 (0.24, 1.38) | 0.006 | 0.73 (0.16, 1.30) | 0.01 | 0.08 (0.01, 0.14) | 0.02 |
|  | Postcentral | 0.81 (0.24, 1.37) | 0.005 | 0.73 (0.17, 1.30) | 0.01 | 0.07 (0.01, 0.14) | 0.03 |
|  | Transverse temporal | 0.80 (0.25, 1.36) | 0.005 | 0.73 (0.17, 1.29) | 0.01 | 0.08 (0.01, 0.14) | 0.02 |
| LH | Rostral middle frontal | 0.80 (0.23, 1.38) | 0.006 | 0.73 (0.15, 1.30) | 0.01 | 0.08 (0.01, 0.14) | 0.03 |
|  | Superior parietal | 0.81 (0.24, 1.37) | 0.005 | 0.73 (0.16, 1.30) | 0.01 | 0.08 (0.01, 0.14) | 0.03 |
|  | Paracentral | 0.81 (0.26, 1.36) | 0.004 | 0.73 (0.18, 1.29) | 0.01 | 0.08 (0.01, 0.14) | 0.03 |
| Surface area |  |  |  |  |  |  |  |
| LH | Rostral middle frontal | 0.81 (0.25, 1.37) | 0.005 | 0.73 (0.17, 1.30) | 0.01 | 0.08 (0.01, 0.14) | 0.03 |

Abbreviations: DQS-8y, diet quality score at eight year-of-age; RH, right hemisphere; LH, left hemisphere; CI confidence interval. The models were adjusted for child sex and age when brain imaging was assessed, maternal education, household income, child ethnic background, child energy intake, child BMI measured at the age of 10 years, maternal diet quality during pregnancy, smoking during pregnancy, alcohol use during pregnancy, folic acid use, and maternal psychopathological symptoms during pregnancy.

Supplemental Table 9 Non-response analysis

|  | Study sample for dietary patterns at one year  (N = 6053) | | Study sample for dietary patterns at eight years  (N = 7254) | |
| --- | --- | --- | --- | --- |
|  | Respondents  (N = 1888) | Non-respondents  (N = 4148) ^1^ | Respondents  (N = 2326) | Non-respondents  (N = 4928) ^1^ |
| Maternal characteristics at enrollment |  |  |  |  |
| Educational level (Low), N (%) | 548 (29.0%) | 1525 (43.1%) | 758 (32.6%) | 2060 (49.5%) |
| Household income per month, N (%) |  |  |  |  |
| < 1200 € | 187 (9.9%) | 516 (16.6%) | 221 (9.5%) | 705 (19.9%) |
| 1200 – 2200 € | 372 (19.7%) | 765 (24.6%) | 512 (22.0%) | 922 (26.1%) |
| > 2200 € | 1329 (70.4%) | 1832 (58.8%) | 1593 (68.5%) | 1910 (54.0%) |
| Alcohol use during pregnancy, N (%) |  |  |  |  |
| Never | 640 (33.9%) | 1468 (44.0%) | 821 (35.3%) | 1955 (48.7%) |
| Until pregnancy was known | 274 (14.5%) | 428 (12.8%) | 342 (14.7%) | 503 (12.5%) |
| Continued | 974 (51.6%) | 1440 (43.2%) | 1163 (50.0%) | 1558 (38.8%) |
| Smoking during pregnancy, N (%) |  |  |  |  |
| Never | 1484 (78.6%) | 2715 (75.5%) | 1840 (79.1%) | 3087 (73.3%) |
| Until pregnancy was known | 187 (9.9%) | 303 (8.4%) | 212 (9.1%) | 344 (8.2%) |
| Continued | 215 (11.4%) | 576 (16.0%) | 274 (11.8%) | 781 (18.5%) |
| Folic acid use, N (%) |  |  |  |  |
| No | 255 (13.5%) | 705 (25.6%) | 391 (16.8%) | 949 (28.7%) |
| Started the first 10 weeks | 598 (31.7%) | 890 (32.3%) | 730 (31.4%) | 1072 (32.5%) |
| Started periconceptional | 1035 (54.8%) | 1161 (42.1%) | 1205 (51.8%) | 1280 (38.8%) |
| Psychopathological symptoms, median (IQR) | 0.1 (0.1, 0.3) | 0.2 (0.1, 0.3) | 0.1 (0.1, 0.3) | 0.2 (0.1, 0.4) |
| Diet quality scores during pregnancy (SD) | 7.9 (1.5) | 7.7 (1.6) | 7.8 (1.5) | 7.6 (1.6) |
|  |  |  |  |  |
| Child characteristics |  |  |  |  |
| Age at the neuroimaging assessment, median (IQR), years | 9.9 (9.7, 10.0) | 10.0 (9.8, 10.6) | 9.9 (9.7, 10.2) | 10.0 (9.8, 10.4) |
| Sex (Girls), N (%) | 974 (51.6%) | 2057 (49.6%) | 1179 (50.7%) | 2440 (49. 5%) |
| Ethnicity |  |  |  |  |
| Dutch | 1346 (71.3%) | 2316 (57.7%) | 1568 (67.4%) | 2584 (54.8%) |
| Non-Dutch Western | 151 (8.0%) | 372 (9.3%) | 223 (9.5%) | 397 (8.4%) |
| Non-Dutch non-Western | 393 (20.8%) | 1326 (33.0%) | 535 (23.0%) | 1733 (36.8%) |
| Diet quality scores, mean (SD) | 4.3 (1.4) | 4.2 (1.4) | 4.5 (1.2) | 4.5 (1.3) |

Abbreviations: IQR, interquartile range; SD, standard deviation. Non-respondents are participants with consent at the time of dietary and neuroimaging measurement, but no (valid) data on dietary data or neuroimaging.

^1^ Non-imputed data are reported for non-respondents.

Supplemental Table 10 Associations of dietary patterns at ages one and eight years with global brain volumes at age 10 years in children with Dutch ethnic background

|  |  | Model 1 |  |  |  | Model 2 |  |
| --- | --- | --- | --- | --- | --- | --- | --- |
|  | *B* | 95% CI | *p* value |  | *B* | 95% CI | *p* value |
| **Total brain volume (cm^3^)** |  |  |  |  |  |  |  |
| *Diet at one year* |  |  |  |  |  |  |  |
| Principal components |  |  |  |  |  |  |  |
| Vegetables, potatoes and grains | 2.40 | -3.22, 8.01 | 0.4025 |  | -1.34 | -7.12, 4.44 | 0.6492 |
| Snacks, processed foods and sugar | -9.01 | -16.61, -1.4 | 0.0203 |  | -6.86 | -14.77, 1.05 | 0.0890 |
| Butter and margarines, whole grains and dairy | 8.53 | 3.12, 13.94 | 0.002 |  | 6.50 | 1.11, 11.89 | 0.0182 |
| DQS-1y | 5.93 | 0.92, 10.93 | 0.0203 |  | 2.64 | -2.42, 7.71 | 0.3062 |
|  |  |  |  |  |  |  |  |
| *Diet at eight years* |  |  |  |  |  |  |  |
| Principal components |  |  |  |  |  |  |  |
| Snacks, potatoes and processed foods | -8.60 | -13.77, -3.43 | 0.0011 |  | **-12.13** | **-19.42, -4.83** | **0.0011*** |
| Fish, vegetables and fruit | 0.38 | -4.34, 5.10 | 0.8739 |  | -2.19 | -7.05, 2.67 | 0.3772 |
| Whole grains, soft fats and dairy | 14.87 | 9.91, 19.82 | 0.0000 |  | **11.16** | **5.58, 16.73** | **0.0001*** |
| Meat replacement, legumes and nuts | -2.22 | -6.89, 2.45 | 0.3516 |  | -3.71 | -8.50, 1.09 | 0.1295 |
| DQS-8y | 7.76 | 3.22, 12.31 | 0.0008 |  | 3.21 | -1.87, 8.29 | 0.2157 |
|  |  |  |  |  |  |  |  |
| **Cerebral white matter volume (cm^3^)** |  |  |  |  |  |  |  |
| *Diet at one year* |  |  |  |  |  |  |  |
| Principal components |  |  |  |  |  |  |  |
| Vegetables, potatoes and grains | 1.34 | -1.26, 3.93 | 0.3118 |  | -0.11 | -2.8, 2.59 | 0.9382 |
| Snacks, processed foods and sugar | -5.23 | -8.74, -1.72 | 0.0035 |  | -4.96 | -8.64, -1.27 | 0.0084 |
| Butter and margarines, whole grains and dairy | 2.99 | 0.48, 5.50 | 0.0194 |  | 2.03 | -0.48, 4.55 | 0.1128 |
| DQS-1y | 3.09 | 0.78, 5.40 | 0.0088 |  | 1.86 | -0.51, 4.23 | 0.1233 |
|  |  |  |  |  |  |  |  |
| *Diet at eight years* |  |  |  |  |  |  |  |
| Principal components |  |  |  |  |  |  |  |
| Snacks, potatoes and processed foods | -3.12 | -5.51, -0.74 | 0.0103 |  | -4.39 | -7.78, -0.99 | 0.0113 |
| Fish, vegetables and fruit | 0.52 | -1.66, 2.69 | 0.6411 |  | -0.26 | -2.52, 2.00 | 0.8240 |
| Whole grains, soft fats and dairy | 5.39 | 3.1, 7.68 | 0.0000 |  | **4.33** | **1.73, 6.92** | **0.0011*** |
| Meat replacement, legumes and nuts | -1.77 | -3.92, 0.38 | 0.1070 |  | -2.16 | -4.39, 0.06 | 0.0570 |
| DQS-8y | 2.60 | 0.5, 4.7 | 0.0151 |  | 1.21 | -1.17, 3.59 | 0.3176 |
|  |  |  |  |  |  |  |  |
| **Cerebral gray matter volume (cm^3^)** |  |  |  |  |  |  |  |
| *Diet at one year* |  |  |  |  |  |  |  |
| Principal components |  |  |  |  |  |  |  |
| Vegetables, potatoes and grains | 0.90 | -2.06, 3.86 | 0.5510 |  | -1.07 | -4.12, 1.99 | 0.4936 |
| Snacks, processed foods and sugar | -3.30 | -7.31, 0.71 | 0.1069 |  | -1.80 | -5.97, 2.37 | 0.3977 |
| Butter and margarines, whole grains and dairy | 4.85 | 2.00, 7.70 | 0.0009 |  | 3.91 | 1.07, 6.76 | 0.0071 |
| DQS-1y | 2.27 | -0.37, 4.91 | 0.0923 |  | 0.57 | -2.1, 3.24 | 0.6752 |
|  |  |  |  |  |  |  |  |
| *Diet at eight years* |  |  |  |  |  |  |  |
| Principal components |  |  |  |  |  |  |  |
| Snacks, potatoes and processed foods | -4.53 | -7.26, -1.8 | 0.0011 |  | **-6.39** | **-10.23, -2.56** | **0.0011*** |
| Fish, vegetables and fruit | -0.4 | -2.89, 2.09 | 0.7543 |  | -1.93 | -4.49, 0.62 | 0.1384 |
| Whole grains, soft fats and dairy | 8.13 | 5.52, 10.74 | 0.0000 |  | **5.75** | **2.82, 8.67** | **0.0001*** |
| Meat replacement, legumes and nuts | 0.02 | -2.44, 2.49 | 0.9856 |  | -0.86 | -3.39, 1.67 | 0.5060 |
| DQS-8y | 4.57 | 2.17, 6.96 | 0.0002 |  | 1.84 | -0.82, 4.50 | 0.1757 |

Abbreviations: DQS-1y, diet quality score at one year-of-age; DQS-8y, diet quality score at eight year-of-age; CI, confidence interval. The effect estimates represent the difference in cubic centimeters for brain volumes per 1 SD higher score on the dietary pattern. Model 1 was adjusted for child sex and age when brain imaging was assessed. Model 2 was additionally adjusted for maternal education, household income, child energy intake, child BMI measured at the age of 10 years, maternal diet quality during pregnancy, smoking during pregnancy, alcohol use during pregnancy, folic acid use, and maternal psychopathological symptoms during pregnancy.

* denotes the associations which remained statistically significant after the Benjamini-Hochberg correction for multiple testing (45 tests) with a FDR ≤ 0.05.

Supplemental Table 11 Association of dietary patterns at ages one and eight years with regional brain volumes at age 10 years in children with Dutch ethnic background

|  |  | Model 1 |  |  |  | Model 2 |  |
| --- | --- | --- | --- | --- | --- | --- | --- |
|  | *B* | 95% CI | *p* value |  | *B* | 95% CI | *p* value |
| **Hippocampal volume (mm^3^)** |  |  |  |  |  |  |  |
| *Diet at one year* |  |  |  |  |  |  |  |
| Principal components |  |  |  |  |  |  |  |
| Vegetables, potatoes and grains | 23.28 | -17.23, 63.78 | 0.2598 |  | 6.78 | -29.9, 43.45 | 0.7171 |
| Snacks, processed foods and sugar | -26.51 | -81.51, 28.49 | 0.3446 |  | 23.20 | -27.39, 73.79 | 0.3684 |
| Butter and margarines, whole grains and dairy | 60.37 | 21.29, 99.46 | 0.0025 |  | 27.02 | -7.17, 61.21 | 0.1213 |
| DQS-1y | 39.41 | 3.27, 75.55 | 0.0326 |  | 6.57 | -25.67, 38.82 | 0.6892 |
|  |  |  |  |  |  |  |  |
| *Diet at eight years* |  |  |  |  |  |  |  |
| Principal components |  |  |  |  |  |  |  |
| Snacks, potatoes and processed foods | -32.62 | -71.54, 6.3 | 0.1004 |  | 22.27 | -25.54, 70.08 | 0.3610 |
| Fish, vegetables and fruit | 2.38 | -33.07, 37.83 | 0.8952 |  | -14.07 | -45.95, 17.8 | 0.3867 |
| Whole grains, soft fats and dairy | 59.72 | 22.22, 97.22 | 0.0018 |  | -3.22 | -39.95, 33.5 | 0.8634 |
| Meat replacement, legumes and nuts | 6.49 | -28.61, 41.6 | 0.7168 |  | 14.95 | -16.46, 46.35 | 0.3507 |
| DQS-8y | 20.87 | -13.35, 55.09 | 0.2317 |  | -34.68 | -67.94, -1.42 | 0.0410 |
|  |  |  |  |  |  |  |  |
| **Amygdala volume (mm^3^)** |  |  |  |  |  |  |  |
| *Diet at one year* |  |  |  |  |  |  |  |
| Principal components |  |  |  |  |  |  |  |
| Vegetables, potatoes and grains | 10.92 | -10.25, 32.09 | 0.3118 |  | 3.68 | -15.58, 22.93 | 0.7080 |
| Snacks, processed foods and sugar | -20.25 | -48.99, 8.48 | 0.1669 |  | 2.08 | -24.31, 28.47 | 0.8773 |
| Butter and margarines, whole grains and dairy | 32.2 | 11.78, 52.63 | 0.0020 |  | 12.61 | -5.32, 30.53 | 0.1679 |
| DQS-1y | 28.36 | 9.5, 47.22 | 0.0032 |  | 12.69 | -4.21, 29.59 | 0.1409 |
|  |  |  |  |  |  |  |  |
| *Diet at eight years* |  |  |  |  |  |  |  |
| Principal components |  |  |  |  |  |  |  |
| Snacks, potatoes and processed foods | -27.68 | -47.07, -8.28 | 0.0052 |  | -12.37 | -36.19, 11.44 | 0.3082 |
| Fish, vegetables and fruit | 3.64 | -14.05, 21.33 | 0.6867 |  | -2.37 | -18.29, 13.55 | 0.7704 |
| Whole grains, soft fats and dairy | 38.2 | 19.53, 56.88 | 0.0001 |  | 10.91 | -7.43, 29.25 | 0.2435 |
| Meat replacement, legumes and nuts | -3.19 | -20.72, 14.33 | 0.7207 |  | 2.66 | -13.11, 18.42 | 0.7411 |
| DQS-8y | 18.53 | 1.47, 35.59 | 0.0333 |  | -3.33 | -19.9, 13.25 | 0.6937 |

Abbreviations: DQS-1y, diet quality score at one year-of-age; DQS-8y, diet quality score at eight year-of-age; CI, confidence interval. The effect estimates represent the difference in cubic centimeters for brain volumes per 1 SD higher score on the dietary pattern. Model 1 was adjusted for child sex and age when brain imaging was assessed. Model 2 was additionally adjusted for maternal education, household income, child ethnic background, child energy intake, child BMI measured at the age of 10 years, maternal diet quality during pregnancy, smoking during pregnancy, alcohol use during pregnancy, folic acid use, and maternal psychopathological symptoms during pregnancy and child intracranial volume.

Supplemental Table 12 Associations of dietary patterns at ages one and eight years with global brain volumes at age 10 years in children without adjustment of diet quality during pregnancy in model 2

|  | *B* | 95% CI | *p* value |
| --- | --- | --- | --- |
| **Total brain volume (cm^3^)** |  |  |  |
| *Diet at one year* |  |  |  |
| Principal components |  |  |  |
| Vegetables, potatoes and grains | -1.10 | -5.66, 3.46 | 0.6351 |
| Snacks, processed foods and sugar | -7.73 | -13.31, -2.15 | 0.0066 |
| Butter and margarines, whole grains and dairy | 3.88 | -0.61, 8.38 | 0.0906 |
| DQS-1y | 2.68 | -1.53, 6.88 | 0.2119 |
|  |  |  |  |
| *Diet at eight years* |  |  |  |
| Principal components |  |  |  |
| Snacks, potatoes and processed foods | -9.09 | -14.61, -3.56 | 0.0013 |
| Fish, vegetables and fruit | -0.94 | -4.87, 2.99 | 0.6404 |
| Whole grains, soft fats and dairy | 9.76 | 5.36, 14.17 | <0.0001 |
| Meat replacement, legumes and nuts | -2.18 | -6.02, 1.67 | 0.2670 |
| DQS-8y | 4.92 | 0.9, 8.94 | 0.0165 |
|  |  |  |  |
| **Cerebral white matter volume (cm^3^)** |  |  |  |
| *Diet at one year* |  |  |  |
| Principal components |  |  |  |
| Vegetables, potatoes and grains | -0.21 | -2.32, 1.9 | 0.8462 |
| Snacks, processed foods and sugar | -4.51 | -7.1, -1.92 | 0.0006 |
| Butter and margarines, whole grains and dairy | 1.04 | -1.04, 3.13 | 0.3262 |
| DQS-1y | 1.52 | -0.43, 3.46 | 0.1272 |
|  |  |  |  |
| *Diet at eight years* |  |  |  |
| Principal components |  |  |  |
| Snacks, potatoes and processed foods | -3.08 | -5.64, -0.51 | 0.0187 |
| Fish, vegetables and fruit | 0.12 | -1.7, 1.95 | 0.8959 |
| Whole grains, soft fats and dairy | 3.26 | 1.2, 5.31 | 0.0019 |
| Meat replacement, legumes and nuts | -1.31 | -3.1, 0.47 | 0.1487 |
| DQS-8y | 1.79 | -0.07, 3.66 | 0.0595 |
|  |  |  |  |
| **Cerebral gray matter volume (cm^3^)** |  |  |  |
| *Diet at one year* |  |  |  |
| Principal components |  |  |  |
| Vegetables, potatoes and grains | -0.77 | -3.18, 1.63 | 0.5288 |
| Snacks, processed foods and sugar | -2.85 | -5.79, 0.09 | 0.0576 |
| Butter and margarines, whole grains and dairy | 2.35 | -0.02, 4.72 | 0.0523 |
| DQS-1y | 0.88 | -1.33, 3.10 | 0.4340 |
|  |  |  |  |
| *Diet at eight years* |  |  |  |
| Principal components |  |  |  |
| Snacks, potatoes and processed foods | -4.86 | -7.77, -1.96 | 0.0010 |
| Fish, vegetables and fruit | -1.12 | -3.18, 0.95 | 0.2889 |
| Whole grains, soft fats and dairy | 5.61 | 3.30, 7.93 | <0.0001 |
| Meat replacement, legumes and nuts | -0.38 | -2.40, 1.63 | 0.7085 |
| DQS-8y | 2.96 | 0.84, 5.07 | 0.0062 |

Abbreviations: DQS-1y, diet quality score at one year-of-age; DQS-8y, diet quality score at eight year-of-age; CI, confidence interval. The effect estimates represent the difference in cubic centimeters for brain volumes per 1 SD higher score on the dietary pattern. Model was adjusted for child sex, age when brain imaging was assessed, maternal education, household income, child ethnic background, child energy intake, child BMI measured at the age of 10 years, smoking during pregnancy, alcohol use during pregnancy, folic acid use, and maternal psychopathological symptoms during pregnancy.

^
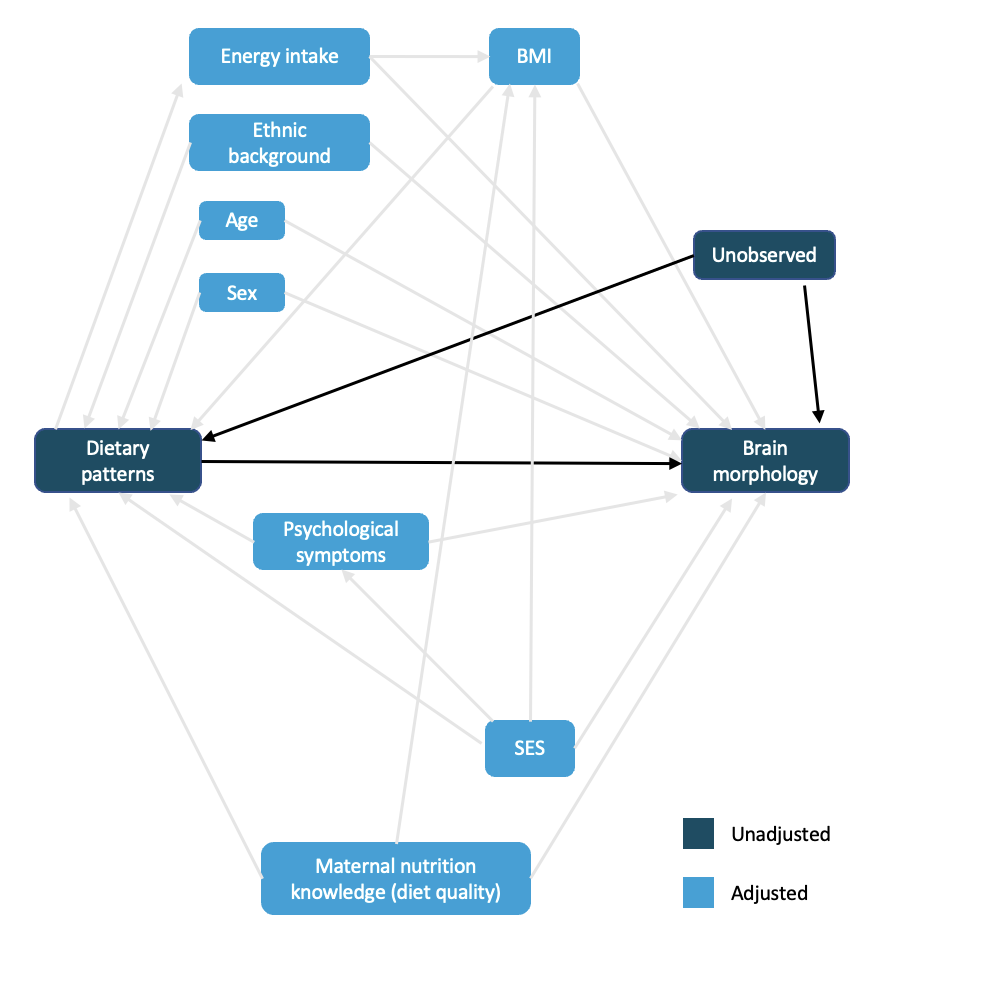
^

Supplemental Fig.1 Simplified directed acyclic graph
